# Validation of JEM Soignances Job-Exposure Matrix Through Comparison with Self-Reported Exposures Among Healthcare Workers in CONSTANCES

**DOI:** 10.1007/s10926-025-10289-0

**Published:** 2025-04-02

**Authors:** Allison Singier, Marc Fadel, Fabien Gilbert, Soignances Group, Yves Roquelaure, Laurent Poiroux, Guillaume Swierczynski, Annette Leclerc, Marcel Goldberg, William Dab, Mohamed Ben Halima, Kevin Jean, Cédric Lemogne, Guillaume Airagnes, Victor Pitron, Pascal Guénel, Marina Kvaskoff, Sandrine Caroly, Ester-MESuRS Collaboration on Occupational Risks, Allison Singier, Alexis Descatha, Marc Fadel, Yves Roquelaure, Laura Temime, William Dab, Mohamed Ben Halima, Kevin Jean, Marie Zins, Laura Temime, Alexis Descatha

**Affiliations:** 1 Univ Angers, Univ Rennes, Inserm, EHESP, Irset (Institut de recherche en santé, environnement et travail)-UMR_S 1085, SFR ICAT, 49000 Angers, France; 2 Univ Angers, CHU Angers, Univ Rennes, Inserm, EHESP, Irset (Institut de recherche en santé, environnement et travail)-UMR_S 1085, SFR ICAT, 49000 Angers, France; 3 Inserm, Université Paris Cité, Université Paris Saclay, Université de Versailles-Saint-Quentin-en-Yvelines (UVSQ), UMS 11 ”Population-based Epidemiological Cohorts Unit”, UMS 011, 94800 Villejuif, France; 4 Modélisation, épidémiologie et surveillance des risques sanitaires (MESuRS), Conservatoire national des arts et métiers, 75003 Paris, France; 5 CHU Angers, Poisoning Control Center, Federation of Prevention, 49000 Angers, France; 6 Department of Occupational Medicine, Epidemiology and Prevention, Hofstra-Northwell Health, Hempstead, NY USA

**Keywords:** Caregiver, Health professional, Job exposure, Occupation, Predictive validity, Accuracy

## Abstract

**Purpose:**

A healthcare-specific job-exposure matrix, JEM Soignances, was recently developed to assess the occupational exposome of healthcare workers. This study aimed to compare estimates of known associations between occupational exposures and health outcomes obtained using JEM Soignances and self-reported data.

**Methods:**

Healthcare professionals from the CONSTANCES cohort with linked data from the French National Healthcare Database (SNDS) were included (*n* = 12219). Exposures were estimated using JEM Soignances (occupations and sectors of activity) and its alternative version (+ establishment size and status), as binary exposure at inclusion or as lifetime cumulative exposure (< 10y/ ≥ 10y). Association with relevant health outcomes (i.e., pain, depressive symptoms, hypertension, cancer, use of psychoactive drugs) were evaluated using logistic regression and compared to estimates obtained from self-reported exposure data in terms of direction, magnitude, and significance.

**Results:**

For organizational exposures, 10/16 associations for JEM Soignances and 12/16 for the alternative version aligned with self-reported data in direction and magnitude. For biomechanical exposures, confidence intervals overlapped in 11/15 associations for JEM Soignances and in 9/15 for the alternative version. For the others, discrepancies generally lean toward underestimation. For psychosocial exposure, JEM Soignances revealed significant limitations: While self-reported effort-reward imbalance was strongly associated with depressive symptoms (aOR = 3.18[2.81;3.59]), JEM Soignances underestimated this association (aOR = 1.37[1.23;1.54]), and the alternative version failed to detect it (aOR = 0.99[0.87;1.13]).

**Conclusion:**

JEM Soignances demonstrated good agreement with self-reported data for organizational and biomechanical exposures but failed for psychosocial exposure, often underestimating or missing associations. Further research is needed to evaluate JEM Soignances validity for biological, chemical, and physical exposures.

**Supplementary Information:**

The online version contains supplementary material available at 10.1007/s10926-025-10289-0.

## Introduction

Healthcare workers play a key role in the effectiveness of the healthcare system, but their activities expose them to numerous occupational hazards, including long working hours, night shifts, physical demands, such as heavy lifting, and various organizational factors [1]. These exposures are associated with a higher incidence of certain health conditions among healthcare workers, including musculoskeletal disorders, mental health issues, and cardiovascular diseases [2–4]. While these health effects are well-documented, most studies tend to focus on short-term, individual exposures, often neglecting cumulative, lifetime occupational exposures due to a lack of comprehensive data.

In this context, job-exposure matrices (JEMs) have been developed as valuable tools for assessing occupational exposures in a standardized and retrospective way, based on job titles and environmental factors [5]. JEMs have been particularly useful for estimating chemical and physical exposures in numerous studies assessing associations with health outcomes, with well-known examples including MATGÉNÉ, FINJEM, and O*NET [6–11].

Recently, a healthcare-specific job-exposure matrix, JEM Soignances, was developed to capture the specific occupational risks faced by healthcare workers [12]. This JEM includes 24 factors covering various facets of healthcare workers’ occupational exposome, such as organizational, biomechanical, physical, biological, chemical, and psychosocial factors. Most of the exposures included in the JEM reported satisfactory performance with moderate to good discriminatory power and fair-to-moderate agreement with individual self-reported data. However, before this tool can be applied in large databases where only job titles and activity sectors are available or by practitioners assessing the occupational hazards encountered by healthcare workers, it is essential to rigorously assess its validity. This study aimed to evaluate whether JEM Soignances can accurately identify occupational exposures among healthcare workers that are known to be associated with health outcomes. To evaluate its validity, associations between occupational exposures estimated using JEM Soignances and relevant health outcomes were estimated and compared with association estimates obtained using self-reported exposure data from the CONSTANCES cohort, which served as the gold standard.

## Methods

### Data Source and Population

The Soignances project relies on the French population-based CONSTANCES cohort. Its data source and population have been described elsewhere [12]. Briefly, CONSTANCES includes approximately 220,000 volunteers recruited between 2012 and 2019, aged 18–69 years, and affiliated with the French general health plan (www.constances.fr) [13, 14]. Among them, the Soignances cohort consists of healthcare workers whose job could be coded based on their response to questionnaires, who had at least 6 months of work experience, and who were currently working at the time of inclusion in the CONSTANCES cohort [12, 15]. For the purposes of this study, data from the French National Healthcare Database (*Système National des Données de Santé*, SNDS) were available for the period 2010–2022; only participants with CONSTANCES data that could be linked to SNDS data were included. The SNDS contains pseudonymized and exhaustive individual information on outpatient healthcare reimbursements (e.g., drug dispensing, physician visits), and medical diagnoses related to long-term diseases (LTD). Additionally, the SNDS includes data from the hospital discharge summary database (*Programme de Médicalisation des Systèmes d’Information*, PMSI), which provides diagnoses coded using the International Classification of Diseases, 10th revision (ICD-10) [16, 17].

### Study Design

In order to compare estimates for associations between healthcare workers’ occupational exposures and the outcomes of interest using JEM Soignances and self-reported data, a cross-sectional study was conducted at the time of inclusion.

### Exposure Measures

The exposures of interest relate to the occupational exposome of healthcare workers, and include 22 factors related to organizational constraints, biomechanical and other physical factors, chemical, and psychosocial factors. These exposures were assessed using two approaches: Self-reported data, which served as the gold standard, and JEM Soignances estimates.

#### Self-Reported Exposures

Exposure data were collected by questionnaire at the time of inclusion in the CONSTANCES cohort (see supplementary material Table 1 for details about exposure assessment). Fifteen exposures were reported as binary variables, while four were assessed using a 4-point Likert scale ranging from ‘never or nearly never’ to ‘always or nearly always’. The level of physical effort at work was assessed for working participants using a scale from 0 (sedentary) to 3 (heavy efforts). Overall intensity of physical effort at work was assessed using the Borg Rating of Perceived Exertion Scale, ranging from 6 (no effort required) to 20 (exhausting). Effort-reward imbalance (ERI) was assessed using a calculated score based on three effort items and seven reward items from the French version of the short ERI questionnaire [18].

**Table 1:** Characteristics and detailed occupational exposures of the Soignances cohort

Characteristic	Missing data (%)	Soignances cohort		Soignances cohort (*n* = 12219)
		Women (*n* = 9,824)	Men (*n* = 2,395)	
*Sociodemographic characteristics*				
Age in years, median [IQR]	0.0	41.0 [32.0;51.0]	40.0 [31.5;51.5]	41.0 [32.0;51.0]
Age group	0.0			
18–29		1,709 (17.4)	434 (18.1)	2,143 (17.5)
30–39		2,823 (28.7)	725 (30.3)	3,548 (29.0)
40–49		2,623 (26.7)	567 (23.7)	3,190 (26.1)
50–59		2,260 (23.0)	493 (20.6)	2,753 (22.5)
≥ 60		409 (4.2)	176 (7.3)	585 (4.8)
Professions	0.0			
Nurses		3,170 (32.3)	474 (19.8)	3,644 (29.8)
Nursing assistants		1,312 (13.4)	172 (7.2)	1,484 (12.1)
Others (medical technicians, assistants, aides)		1,210 (12.3)	207 (8.6)	1,417 (11.6)
Physiotherapists, rehabilitation specialists		979 (10.0)	351 (14.7)	1,330 (10.9)
Physicians		726 (7.4)	414 (17.3)	1,140 (9.3)
Hospital service agents		379 (3.9)	109 (4.6)	488 (4.0)
Clinical managers		358 (3.6)	71 (3.0)	429 (3.5)
Psychologists, psychotherapists		313 (3.2)	58 (2.4)	371 (3.0)
Pharmacists		256 (2.6)	77 (3.2)	333 (2.7)
Pharmacy technicians		307 (3.1)	22 (0.9)	329 (2.7)
Interns (medicine, dentistry, pharmacy)		175 (1.8)	127 (5.3)	302 (2.5)
Midwives^a^				283 (2.3)
Dentists		119 (1.2)	115 (4.8)	234 (1.9)
Opticians, audiologists, medical equipment specialists		144 (1.5)	80 (3.3)	224 (1.8)
Ambulance drivers		64 (0.7)	90 (3.8)	154 (1.3)
Veterinarians		38 (0.4)	19 (0.8)	57 (0.5)
Years of experience by PCS and NAF, median [IQR]	0.3	11.0 [5.0;20.0]	11.0 [5.0;20.0]	11.0 [5.0;20.0]
*Medical characteristics*				
BMI, median [IQR]	1.3	23.0 [20.8;26.1]	24.1 [22.2;26.6]	23.3 [21.1;26.2]
BMI categories	1.3			
Underweight		407 (4.2)	36 (1.5)	443 (3.7)
Normal		6,161 (63.5)	1,390 (58.8)	7,551 (62.6)
Overweight		2,139 (22.0)	761 (32.2)	2,900 (24.0)
Obesity		690 (7.1)	146 (6.2)	836 (6.9)
Severe obesity		304 (3.1)	32 (1.4)	336 (2.8)
Limitations in daily activity				
Significant pain	1.2	1,110 (11.4)	177 (7.5)	1,287 (10.7)
Significant fatigue	1.2	1,193 (12.3)	137 (5.8)	1,330 (11.0)
Sleep disorders	1.2	700 (7.2)	88 (3.7)	788 (6.5)
Depressive symptoms	1.2	301 (3.1)	50 (2.1)	351 (2.9)
Use of psychoactive drugs (6 months following inclusion)				
Antidepressants	0.0	712 (7.2)	107 (4.5)	819 (6.7)
Anxiolytics	0.0	788 (8.0)	105 (4.4)	893 (7.3)
Hypnotics	0.0	292 (3.0)	72 (3.0)	364 (3.0)
Overall pain (intensity ≥ 6)	15.0	2,358 (28.0)	339 (17.6)	2,697 (26.1)
Pain location (intensity ≥ 6)				
Neck	13.0	871 (10.1)	104 (5.3)	975 (9.2)
Shoulder	14.0	722 (8.4)	90 (4.6)	812 (7.7)
Elbow/forearm	15.0	266 (3.2)	29 (1.5)	295 (2.8)
Hand/wrist/fingers	14.0	486 (5.7)	54 (2.8)	540 (5.2)
Lower back	13.0	1,238 (14.4)	173 (8.7)	1,411 (13.3)
Knee/leg	14.0	645 (7.6)	114 (5.8)	759 (7.2)
Hypertension	0.1	1,463 (14.9)	637 (26.6)	2,100 (17.2)
CESD Score, median [IQR]	3.2	8.0 [4.0;14.0]	6.0 [3.0;11.0]	8.0 [4.0;13.0]
Depressive symptoms (CES-D ≥ 19)	3.2	1,430 (15.1)	203 (8.7)	1,633 (13.8)
History of cancer	0.0	463 (4.7)	58 (2.4)	521 (4.3)
Cancer (follow-up)	5.3	820 (8.9)	150 (6.5)	970 (8.4)
*Occupational exposures at baseline*				
* Organizational constraints*				
Late hours (bedtime after midnight)	0.9	880 (9.0)	325 (13.7)	1,205 (10.0)
Early hours (up before 5am)	0.9	711 (7.3)	186 (7.8)	897 (7.4)
Sleepless nights	0.6	980 (10.0)	252 (10.6)	1,232 (10.1)
Long working hours (> 10 h)	0.8	2,167 (22.3)	881 (36.9)	3,048 (25.1)
Weekly rest < 48 h consecutive	1.3	1,456 (15.0)	430 (18.1)	1,886 (15.6)
Shift work	1.4	2,183 (22.5)	391 (16.5)	2,574 (21.4)
Saturday work (more than one in two)	0.6	2,226 (22.8)	523 (22.0)	2,749 (22.6)
Sunday work (more than one in two)	0.8	1,626 (16.7)	300 (12.6)	1,926 (15.9)
* Biomechanical factors*				
Time-constrained job	0.8	225 (2.3)	70 (2.9)	295 (2.4)
Repetitive work	3.7	2,544 (26.9)	541 (23.3)	3,085 (26.2)
Physically difficult work	1.5	1,950 (20.2)	350 (14.7)	2,300 (19.1)
Physical effort at work	4.1	1,409 (15.0)	305 (13.1)	1,714 (14.6)
Carry heavy loads	1.3	2,480 (25.6)	508 (21.3)	2,988 (24.8)
Carry heavy loads (> 25 kg)	4.1	1,354 (14.4)	262 (11.3)	1,616 (13.8)
Arms above shoulder	2.3	1,008 (10.5)	100 (4.3)	1,108 (9.3)
Kneel or squat	1.6	3,706 (38.3)	507 (21.5)	4,213 (35.1)
Intense physical effort (Borg)	2.4	5,047 (52.7)	995 (42.6)	6,042 (50.7)
* Physical and chemical factors*				
Noise pollution	0.6	939 (9.6)	168 (7.0)	1,107 (9.1)
Noisy tools	0.8	582 (6.0)	187 (7.9)	769 (6.3)
Ionizing radiation	4.7	1,053 (11.2)	443 (19.4)	1,496 (12.8)
Formaldehyde	3.9	263 (2.8)	62 (2.7)	325 (2.8)
* Psychosocial factor*				
High effort-reward imbalance (≥ 1.5)	3.6	1,564 (16.5)	257 (11.1)	1,821 (15.5)
*Cumulative time of occupational exposures at baseline (≥ 10y)*				
* Organizational constraints*				
Late hours (bedtime after midnight)	0.9	753 (7.7)	287 (12.1)	1,040 (8.6)
Early hours (up before 5am)	0.9	658 (6.8)	170 (7.2)	828 (6.8)
Sleepless nights	0.6	883 (9.0)	216 (9.1)	1,099 (9.1)
Long working hours (> 10 h)	0.8	1,416 (14.5)	596 (25.0)	2,012 (16.6)
Weekly rest < 48 h consecutive	1.3	1,180 (12.2)	361 (15.2)	1,541 (12.8)
Shift work	1.4	1,889 (19.5)	295 (12.4)	2,184 (18.1)
Saturday work (more than one in two)	0.6	1,988 (20.4)	489 (20.5)	2,477 (20.4)
Sunday work (more than one in two)	0.8	1,442 (14.8)	264 (11.1)	1,706 (14.1)
* Biomechanical factors*				
Time-constrained job	0.8	172 (1.8)	52 (2.2)	224 (1.8)
Physically difficult work	1.5	1,729 (17.9)	308 (13.0)	2,037 (16.9)
Carry heavy loads	1.3	2,154 (22.2)	415 (17.4)	2,569 (21.3)
* Physical and chemical factors*				
Noise pollution	0.6	610 (6.2)	109 (4.6)	719 (5.9)
Noisy tools	0.8	422 (4.3)	146 (6.1)	568 (4.7)
Ionizing radiation	4.7	824 (8.8)	330 (14.5)	1,154 (9.9)
Formaldehyde	3.9	269 (2.9)	58 (2.5)	327 (2.8)

Data are *n* (%), unless otherwise specified; %: percentage, PCS: *Professions et catégories sociales*, NAF: *Nomenclature des activités françaises*, IQR: Interquartile Range, CES-D: Center for Epidemiologic Studies Depression Scale

^a^In order to maintain data confidentiality, n (%) for sex stratification are not displayed when at least one group contains fewer than 11 individuals

All ordinal and continuous exposures were dichotomized. For the 4-point Likert scale variables, ‘often’ and ‘always or nearly always’ were considered to indicate exposure. Participants reporting heavy physical efforts were considered exposed, while for the Borg Rating and the ERI, values higher or equal to 13 and 1.5 were considered indicative of intense physical effort and high effort-reward imbalance, respectively.

When available, the cumulative time of exposure was determined as the number of years of exposure reported in the questionnaire and was dichotomized as ≥ 10 years / < 10 years of exposure***,*** in line with the WHO/ILO Technical Advisory Group recommendations and previous occupational epidemiology studies [19, 20].

#### JEM Soignances-Estimated Exposures

JEM Soignances is a job-exposure matrix focused on occupational risk factors for healthcare workers. Developed using CONSTANCES self-reported data, this JEM provides frequency of exposure to organizational constraints, biomechanical, physical, chemical, biological, and psychosocial factors. Three versions of the JEM are available: The main version, which is based on the French classification of occupations (*Professions et catégories sociales*, PCS 2003) and sectors of activity according to the French activities coding (*Nomenclature des activités françaises*, NAF 2008) [21, 22]; a simplified version using only PCS data; and an alternative version incorporating PCS, NAF, health establishment status (public or private), and size (< 200 or ≥ 200 employees) [12].

Here, exposures were estimated using the main and alternative versions of JEM Soignances according to the jobs held at the time of inclusion in CONSTANCES (see Supplementary material 2 for summary of exposures included in JEM Soignances by occupational groups). Frequencies of exposure were then dichotomized using the optimal cut-off reported by the authors [12].

For the cumulative exposure assessment, the number of years spent in an exposed job was used to estimate the total years of exposure and dichotomized as ≥ 10 years / < 10 years of exposure.

### Outcome Measures

Using CONSTANCES and SNDS data, multiple outcomes were measured at the time of inclusion (except for cancer and psychoactive drug use which were assessed using SNDS data after inclusion; see supplementary material Table 1 for detailed information on outcome measures). For organizational exposures, associations with limitations in daily activity due to sleep disorders and depressive symptoms, measured using the Center of Epidemiologic Studies-Depression Scale (CESD score ≥ 19) [23], were estimated. For biomechanical exposures, associations with overall pain and specific musculoskeletal pain (e.g., hand/ wrist/fingers, neck, shoulder, elbow/forearm, lower back, knee/leg) were estimated. Overall pain was defined as the presence of pain at any location with an intensity of ≥ 6/10. For physical and chemical exposures, associations between noise pollution and noisy tools and hypertension and depressive symptoms were estimated. For exposure to ionizing radiation and formaldehyde, associations with cancer were estimated. Finally, for psychosocial exposure (high effort-reward imbalance), associations with depressive symptoms, and use of medications, such as antidepressants, anxiolytics and hypnotics in the 6 months following inclusion were estimated.

### Data Analysis

Associations between exposures at inclusion and outcomes of interest were assessed using logistic regression. Three models were performed for each analysis: A crude model, a model adjusted for sex and age, and a model adjusted for age and stratified by sex. For associations between biomechanical exposures and pain, BMI was also considered for adjustment. Odds ratios (OR) and their 95% confidence intervals (95% CI) were graphically represented using forest plots to facilitate comparisons between association estimates based on self-reported exposures and JEM-estimated exposures using both main and alternative JEM. Differences in direction, strength of association, precision, and statistical significance of estimates were investigated.

Secondary analyses were performed to assess associations between cumulative years of exposure (≥ 10 years/ < 10 years) and outcomes of interest using the same methodology as described above.

Statistical analyses were performed using R software (v 4.3.2, R Foundation for Statistical Computing, Vienna, Austria).

## Results

### Population

**Fig. 1:**
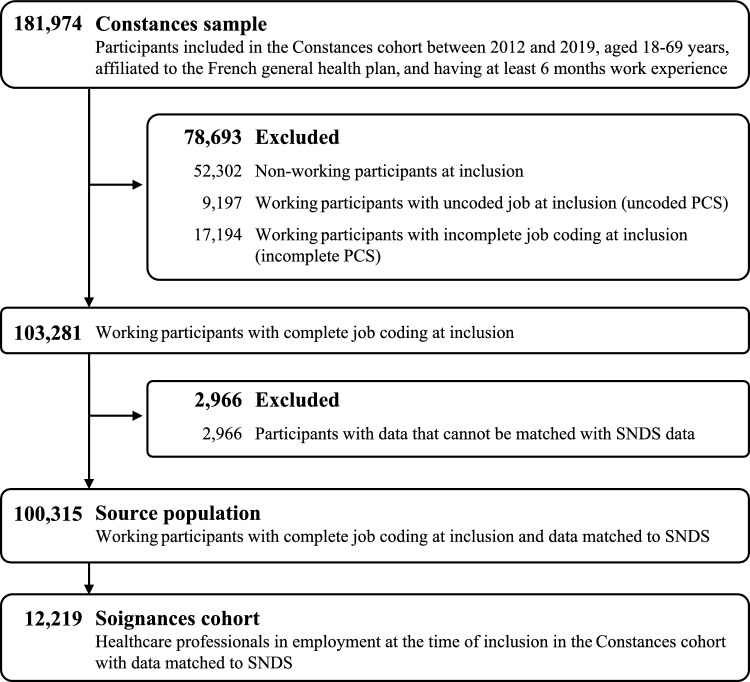
Flowchart of study population selection. PCS: *Professions et catégories sociales,* SNDS: French National Healthcare Database (*Système National des Données de Santé*)

Among the 181,974 participants with a minimum of 6 months of work experience included in the CONSTANCES cohort, 100,315 were active with complete job coding at the time of inclusion and had data that could be linked to the SNDS (Fig. 1). Among them, 12219 healthcare workers were identified and included in the Soignances cohort.

As described elsewhere, this population was constituted of a majority of women (80.4%) and had a median age of 41 years (IQR: 32–51; Table 1). The most represented professions among women were nurses (32.3%), nursing assistants (13.4%), medical technicians, assistants, and aides (12.3%), and physiotherapists and rehabilitation specialists (10.0%). Among men, the most represented professions were nurses (19.8%), physicians (17.3%), and physiotherapists and rehabilitation specialists (14.7%). While overweight and obesity affected 22.0% and 10.2% of women, respectively, these prevalences were of 32.2% and 7.6% for men. Almost 5% of women (4.7%) had a history of cancer at inclusion, twice as frequent as in men (2.4%), and during follow-up, nearly 1 in 10 women (8.9%) were diagnosed with cancer, compared with 6.5% of men. Nearly 30% of women reported suffering from pain in at least one area of the body, compared to less than 20% of men. Among women, the most common pain locations were lower back and neck, while for men, they were lower back and knee/leg. Women were also more likely than men to report limitations in daily activities due to sleep disorders (7.2 vs. 3.7%) and depressive symptoms (15.1 vs. 8.7%). Additionally, the use of psychoactive drugs in the six months following inclusion was higher among women, with 3.0% using hypnotics, 8.0% using anxiolytics, and 7.2% using antidepressants, compared to 3.0%, 4.4%, and 4.5% for men, respectively. Conversely, hypertension was more prevalent in men, affecting 26.6% compared to 14.9% of women.

### Organizational Exposures and Sleep Disorders/Depressive Symptoms

**Fig. 2:**
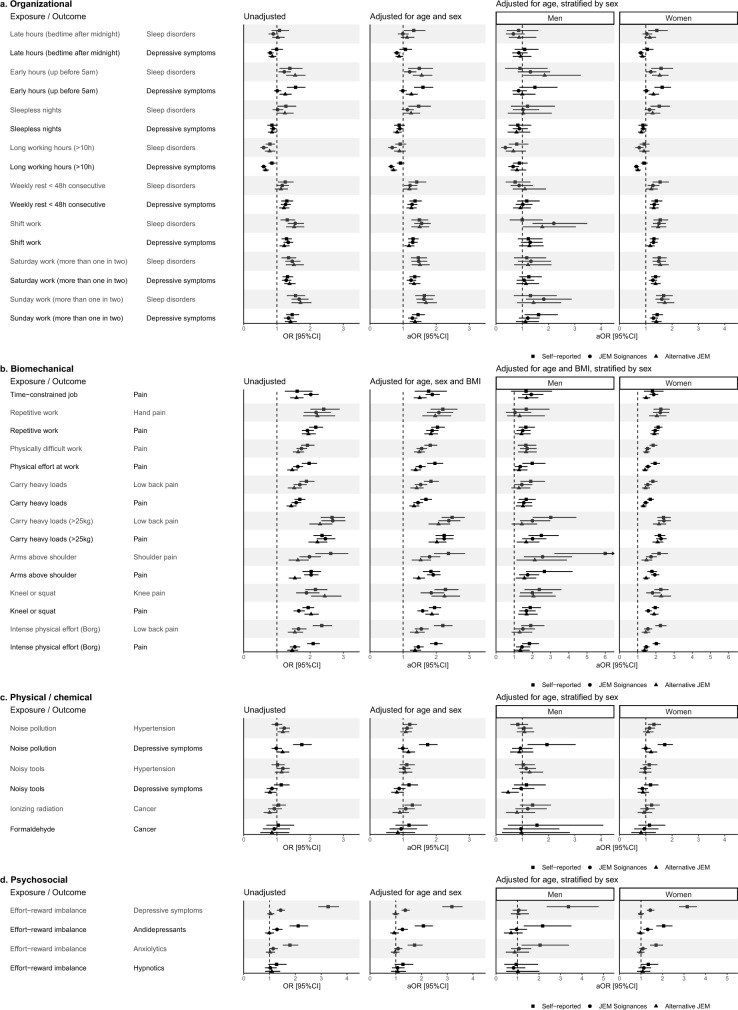
Forest plot of association estimates for all tested associations with occupational exposures at baseline (a. Organizational, b. Biomechanical, c. Physical/chemical, d. Psychosocial). aOR: Adjusted Odds Ratio, OR: Odds Ratio, 95%CI: 95% confidence interval

Figure 2a describes associations between organizational constraints and sleep disorders or depressive symptoms (supplementary material Table 2 for detailed estimates). The direction and magnitude of associations remained consistent with self-reported exposure method for 10 of the 16 tested associations for JEM Soignances and 12/16 for the alternative version, all of which were statistically significant. For the remaining associations, differences were observed regarding direction, magnitude or significance, as for, in adjusted models, late hours and sleep disorders (self-reported: aOR [95%CI] = 1.32 [1.03;1.67], JEM Soignances: 0.99 [0.84;1.17], alternative JEM: 1.12 [0.93;1.34]), early hours and depressive symptoms (1.60 [1.33;1.91], 0.99 [0.89;1.11], 1.25 [1.08;1.44]), sleepless night and sleep disorders (1.47 [1.16;1.83], 1.13 [0.96;1.32], 1.24 [1.02;1.51]), late hours and depressive symptoms (1.07 [0.89;1.27], 0.81 [0.72;0.92], 0.88 [0.77;1.00]), or long working hours and depressive symptoms (0.92 [0.81;1.04], 0.64 [0.56;0.74], 0.71 [0.62;0.82]). These differences persisted after stratification, though estimates were less precise for men in the sex-stratified models due to smaller sample sizes.

**Fig. 3:**
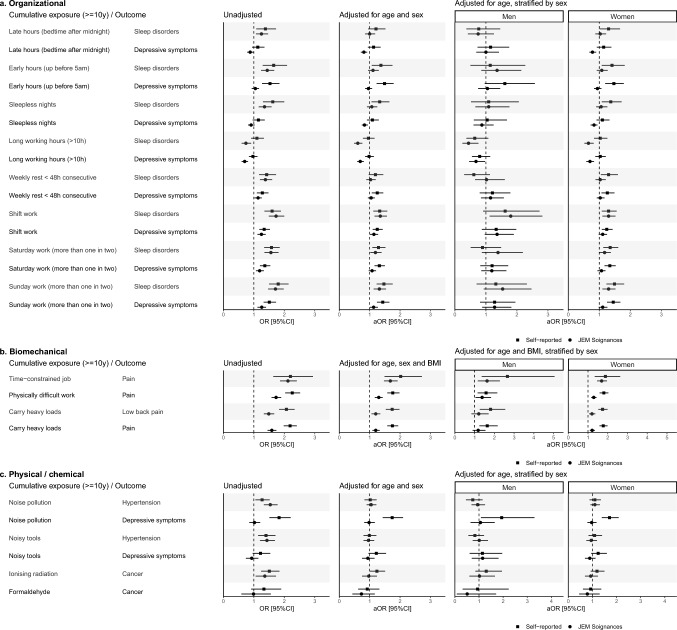
Forest plot of association estimates for all tested associations with cumulative occupational exposures (≥ 10 years) at baseline (a. Organizational, b. Biomechanical, c. Physical/chemical). aOR: Adjusted Odds Ratio, OR: Odds Ratio, 95%CI: 95% confidence interval

Considering cumulative exposure to organizational constraints, JEM Soignances demonstrated lower performance in estimating associations compared to exposure at inclusion, showing greater discrepancies in terms of direction, magnitude, and significance of the associations with those obtained using self-reported data (Fig. 3a).

### Biomechanical Exposures and Pain

For associations between biomechanical exposures and pain, Fig. 2b shows that, except for the stratified model in men, all associations identified with self-reported exposures were also observed using both JEMs (supplementary material Table 3 for detailed estimates). Of the 15 associations, 11 had overlapping confidence intervals between self-reported exposures and JEM Soignances-based exposures, while 9 showed overlap with the alternative version. Discrepancies were generally low, tending toward underestimation when using JEM-based estimates, such as physical effort at work and overall pain (self-reported: aOR [95%CI] = 1.96 [1.73;2.21], JEM Soignances: 1.52 [1.38;1.68], alternative JEM: 1.38 [1.23;1.54]), intense physical effort and low back pain (2.20 [1.94;2.49], 1.55 [1.35;1.77], 1.41 [1.21;1.65]), and intense physical effort and overall pain (1.99 [1.81;2.19], 1.46 [1.32;1.62], 1.36 [1.21;1.54]). Overall, compared to the other exposure estimation methods, the alternative JEM seemed to underestimate these associations. These trends persisted after stratification by sex.

Considering cumulative exposure to biomechanical factors, discrepancies between associations measured using self-reported data and JEM Soignances were more pronounced compared to exposure at inclusion (Fig. 3b). The strength of associations was underestimated using JEM Soignances for physically difficult work and overall pain (self-reported: Aor [95%CI] = 1.76 [1.58;1.98], JEM Soignances: 1.30 [1.17;1.43]), carrying heavy loads and low back pain (1.74 [1.53;1.98], 1.20 [1.06;1.35]), and carrying heavy loads and overall pain (1.75 [1.58;1.94], 1.20 [1.09;1.33]).

### Physical/Chemical Exposures and Hypertension/Depressive Symptoms/Cancer

In the adjusted models, significant associations were found for only three of the six tested associations involving self-reported physical and chemical exposures (i.e., noisy tools—hypertension, noisy tools—depressive symptoms, formaldehyde—cancer; Fig. 2c, supplementary material Table 3), while none of these associations were significant when using JEMs. For both the association between noise pollution and depressive symptoms and ionizing radiation and cancer, no association were found using JEM estimates (self-reported: aOR [95%CI] = 1.74 [1.48;2.04], JEM Soignances: 0.99 [0.84;1.15], alternative JEM: 1.16 [0.98;1.36] and 1.28 [1.04;1.56], 1.08 [0.85;1.35], 0.90 [0.68;1.17], respectively). Stratifying for sex did not improve the results.

As with exposure at inclusion, JEM Soignances did not detect any association between cumulative exposure to noise pollution and depressive symptom (self-reported: aOR [95%CI] = 1.74 [1.43;2.10], JEM Soignances: 0.98 [0.82;1.17]; Fig. 3c). For the five other associations assessed, estimates were generally comparable across models (i.e., unadjusted, adjusted for age and sex, adjusted for age and stratified by sex), with overlapping confidence intervals between self-reported exposures and JEM-based estimates.

### Psychosocial Exposure and Depressive Symptoms/Psychoactive Drugs Use

For exposure to effort-reward imbalance, associations with the use of antidepressants and anxiolytics were largely underestimated or not detected when using JEMs estimates (Fig. 2d, supplementary material Table 3). Similarly, while self-reported high effort-reward imbalance was strongly associated with depressive symptoms after adjustment for age and sex (aOR [95% CI] = 3.18 [2.81;3.59]), the estimates from JEM Soignances were largely underestimated (1.37 [1.23;1.54]) and the alternative JEM Soignances (0.99 [0.87;1.13]) failed to find this association. In the stratified model, these discrepancies were even more pronounced among men.

## Discussion

### Principal Findings

This study aimed to assess the validity of JEM Soignances, a JEM specific to healthcare workers, by comparing association estimates based on JEM-derived exposures from two versions of this JEM with self-reported exposure data considered as a gold standard. Our findings suggest that both the main and alternative versions of JEM Soignances, when inaccurate, generally tend to underestimate associations with various outcomes compared to self-reported data. For organizational factors, JEM Soignances provided better results than expected regarding the internal validity performance previously observed (good sensitivity, poor specificity) [12]. Performance for biomechanical factors was good, aligning with internal validity measures. In contrast, psychosocial factors were poorly captured by JEM estimates. This result was expected, given the challenge of assessing the subjective nature of psychosocial factors through JEMs and the previously observed poor internal validity for these factors. Assessing the validity of physical and chemical factors was more complex due to the long-term nature of the outcomes (e.g., cancer), and potential for reverse associations (e.g., noise pollution and depressive symptoms). Consequently, definitive conclusions regarding the validity of JEM Soignances for these exposures cannot yet be drawn. The alternative version of JEM Soignances did not outperform the main version, except for organizational factors. This improved performance for organizational exposures may reflect the influence of contextual variables, such as establishment size and status, which are particularly relevant for refining exposure assessment to these factors*.*

Although JEMs are often considered efficient tools for assessing lifetime exposure, our study showed greater discrepancies between JEM estimates and self-reported data when considering cumulative lifetime exposure compared to binary exposure at inclusion. While the performance for biomechanical exposures remained relatively consistent, the results for organizational factors were more variable. These findings underscore the importance of evaluating JEMs across different conditions and exposure scenarios to have a better understanding of their validity.

### Comparison with the Literature

The results of the current study align with existing literature, which often reports that JEMs tend to show strong validity for biomechanical exposures and their association with pain. For instance, a Finnish JEM focused on physical risk factors in low back pain demonstrated good validity, even though JEM association estimates were less precise than those of self-reported data due to a potential non-differential misclassification bias [24]. Similarly, a Norwegian JEM focused on mechanical and psychosocial exposures reported good overall performance, despite an underestimation of certain associations, such as those involving heavy physical work [25]. Additionally, the French gendered CONSTANCES JEM, focused on physical risk factors, showed good agreement with self-reported data but highlighted that its gendered version did not outperform non-gendered CONSTANCES JEM [26]. Wuytack et al. further suggest that stratification by sex can, in some cases, be as effective as gendered JEMs for assessing occupational exposures [26]. However, in our study, when using JEM-based estimates with sex stratification, it appears to modify certain associations, such as the association between shift work and sleep disorders, particularly in men. It could result from the underrepresentation of men in the Soignances cohort, which may lead to over- or underestimation of exposure levels. While generally non-differential, this classification error can distort associations, especially when the number of events is low.

The poor performance for psychosocial exposures, that could be due to their subjective nature, is also consistent with previous findings. For example, a French JEM based on the SUMER survey [27] and a Finnish JEM for psychosocial factors [28] both reported highly underestimated or even reversed associations for outcomes like poor perceived health and depressive symptoms. While Niedhammer et al. suggested that incorporating company size could improve validity for psychosocial exposures [29], our study did not confirm this finding in the healthcare workers population, as the alternative version of JEM Soignances did not outperform the main version.

In contrast to biomechanical and psychosocial exposures, organizational and physical exposures remain less studied in the context of JEMs, and to our knowledge, their validity has not been extensively assessed. This limits the ability to compare our findings with external data.

### Strengths and Limitations

This study evaluated the validity of JEM Soignances by assessing known associations between occupational exposures included in the JEM and health outcomes. Specifically, it compared association estimates derived from JEM-based exposure data and self-reported exposures across different models: (i) crude, (ii) adjusted for age and sex (and BMI for biomechanical exposures), and (iii) adjusted for age (and BMI for biomechanical exposures) and stratified by sex. These comparisons were conducted using the JEM to estimate exposures both at inclusion and cumulatively over a lifetime. Alongside prior research focused on the development of the JEM Soignances and its internal validity [12], this study provides a comprehensive view of its validity across different contexts of use. This study benefits from the use of a large cohort of healthcare professionals within the CONSTANCES cohort. These data allow for a robust assessment of occupational exposures with minimal missing data.

However, this study has limitations. First, due to lack of data on health professionals in France, the current study was conducted using the same dataset as that used for the development of the JEM, which may have led to an overestimation of its performance. Furthermore, CONSTANCES data relies on voluntary participation, rendering it difficult to ensure the representativeness of our sample compared to the broader population of healthcare workers in France. Second, the underrepresentation of men in the Soignances cohort may lead to imprecise estimation of exposures and distort associations in sex-stratified models. Addressing this imbalance could involve creating a gender-specific version of the JEM, though this approach may exclude certain occupational groups due to the limited number of male participants. Third, while this study tests the JEM in diverse contexts, it does not address whether the JEM remains valid for other countries or time periods beyond the inclusion period (2012–2019), such as the post-COVID period. Fourthly, no conclusions can be drawn for certain associations with long-term outcomes, such as cancer, which cannot be reliably estimated due to the insufficient follow-up period. Yet, this limitation only applies to exposure to formaldehyde and ionizing radiation. Fifth, the statistical analysis was not structured to allow for formal statistical comparisons of ORs derived from self-reported data and JEM-based estimates. Nonetheless, the methodology used is widely employed in occupational epidemiology to assess JEM validity [24–28].

Finally, like all JEMs, JEM Soignances may underestimate associations due to non-differential misclassification bias, which reduces precision. This occurs because JEMs assign the same exposure level to all individuals within a given occupational group, leading to misclassification when there is high variability within the group. Since this misclassification is generally non-differential regarding health outcomes, it tends to reduce the strength of observed associations. However, estimates obtained using JEMs are less biased than those based on self-reported data which are subject to memory biases, particularly in recalling lifetime exposures and detailed exposure periods. These biases may lead to overestimations of associations, especially among symptomatic [5, 30, 31]. It is thus possible that in the secondary analysis, which focused on cumulative exposures, self-reported data—used as the gold standard—may have provided less accurate association estimates than JEM-based estimates. Furthermore, for cumulative exposures, the healthy worker survivor effect may have influenced both self-reported and JEM-based exposure estimates. Workers with prolonged exposure might become more tolerant to the conditions and report fewer health events, while those who changed jobs due to health issues related to occupational exposures could be underrepresented, as only health professionals were included. This selection bias could lead to an underestimation of the associations between long-term exposure and health outcomes and may explain some results suggesting that long-term exposures appeared protective.

## Conclusion

JEM Soignances demonstrated a strong alignment with self-reported data, particularly for organizational and biomechanical factors, with results globally aligning with internal validity measures. However, JEM Soignances estimates for cumulative exposures were less closely aligned with self-reported data. Furthermore, its application to certain exposures, such as psychosocial factors, frequently led to underestimation of associations in the best scenarios and, in the worst cases, failed to identify existing associations or suggested spurious ones when compared to self-reported data. Sex-stratified analyses produced imprecise exposure estimates for male healthcare workers, likely reflecting their underrepresentation in the cohort. These findings highlight both the potential and the limitations of JEM Soignances and emphasize the importance of ongoing validation of JEMs across diverse contexts and populations.

## Supplementary Information

Below is the link to the electronic supplementary material.

Supplementary file1 (DOCX 115 KB)

Supplementary file2 (XLSX 29 KB)

## Data Availability

As the access to CONSTANCES and SNDS data is highly regulated, data cannot be shared publicly. Details on the procedure for accessing these data are available at: https://www.constances.fr/en/scientific-area/access-to-constances-2/.
