# Supplementary material for: Validation of JEM Soignances Job-Exposure Matrix Through Comparison with Self-Reported Exposures Among Healthcare Workers in CONSTANCES
Source: J Occup Rehabil. 2025 Apr 2;36(2):480–92. doi: 10.1007/s10926-025-10289-0 (PMC13099736; doi:10.1007/s10926-025-10289-0)
Supplement: Supplementary file 1 — Supplementary file1 (DOCX 115 KB) [file 10926_2025_10289_MOESM1_ESM.docx]

**SUPPLEMENTARY MATERIAL 1**

**Validation of JEM Soignances job-exposure matrix through comparison with self-reported exposures among healthcare workers in CONSTANCES**

Allison Singier, Marc Fadel, Fabien Gilbert, Soignances group, Ester-MESuRS collaboration on occupational risks, Marie Zins, Laura Temime, Alexis Descatha

**Supplementary material table 1. Details about the identification of outcomes and self-reported exposures**

| **Variables** | **Identification ^a^** |
| --- | --- |
| **Outcomes of interest (CONSTANCES questionnaires and SNDS data)** | |
| Depressive symptoms | *Calculated* – CES-D score ≥ 19 (CONSTANCES questionnaire at inclusion) |
| Limitations in daily activity due to sleep disorders | Over at least the past 6 months, have you been limited, i.e. do you experience difficulties due to a health-related problem, in performing routine activities (at home, at work, during leisure activities, etc.) by comparison to other people of your age? Y/N  If yes, for what reasons? Sleep disorders (CONSTANCES questionnaire at inclusion) |
| Pain   - neck, - shoulder, - elbow/forearm, - hand/wrist/fingers, - lower back, - knee/leg | Intensity of pain ≥ 6 according to the following questions (CONSTANCES questionnaire at inclusion):  Over the past 7 days, have you experienced any problems (aches, pains, discomfort, numbness) in the following areas of the body? Y/N  Neck, Shoulder, Elbow / forearm, Hand / Wrist / Fingers, Lower back, Knee / Leg  If yes, how would you rate the intensity of this(these) problem(s) according to the scale below? 0 (no discomfort or pain) – 10 (maximum conceivable pain) |
| Hypertension | Systolic blood pressure ≥140 mmHg and/or diastolic blood pressure ≥90 mmHg (CONSTANCES paraclinical data collection at inclusion) **or** reported hypertension with recorded age of diagnosis (CONSTANCES medical questionnaire) **or** ≥ 1 dispensing of antihypertensive drugs in the 6 months prior to inclusion (SNDS data); ATC codes: C03EA, C02AB02, C02AC01, C02AC02, C02AC05, C02AC06, C02CA01, C02CA06, C02DC01, C02LA01, C03AA01, C03AA03, C03BA04, C03BA10, C03BA11, C03BX03, C03CA01, C03CA02, C03CA03, C03DA01, C03DB01, C03EA01, C03EA04, C07AA02, C07AA03, C07AA05, C07AA06, C07AA12, C07AA15, C07AA16, C07AA23, C07AB02, C07AB03, C07AB04, C07AB05, C07AB07, C07AB08, C07AB12, C07AG01, C07BA02, C07BB02, C07BB03, C07BB07, C07BB12, C07CA03, C07DA06, C07FB02, C07FB03, C08CA01, C08CA02, C08CA03, C08CA04, C08CA05, C08CA08, C08CA09, C08CA11, C08CA13, C08CX01, C08DA01, C08DB01, C08GA02, C09AA01, C09AA02, C09AA03, C09AA04, C09AA05, C09AA06, C09AA07, C09AA08, C09AA09, C09AA10, C09AA13, C09AA15, C09AA16, C09BA01, C09BA02, C09BA03, C09BA04, C09BA05, C09BA06, C09BA07, C09BA09, C09BA15, C09BB02, C09BB04, C09BB10, C09BX02, C09CA01, C09CA02, C09CA03, C09CA04, C09CA06, C09CA07, C09CA08, C09DA01, C09DA02, C09DA03, C09DA04, C09DA06, C09DA07, C09DA08,C09DB01, C09DB02, C09DB04, C09XA02, C09XA52, C10BX03 |
| History of cancer | Long-term disease in relation to cancer (SNDS data, 2 years prior to inclusion; ICD-10 codes: C00-D48) **or** hospitalization with main or related diagnosis in relation to cancer (SNDS data, 2 years prior to inclusion; ICD-10 codes: C00-D48, Z51.0 - radiotherapy, Z51.1 - chemotherapy) |
| Cancer (follow-up) | Hospitalization during follow-up with main diagnosis in relation to cancer (SNDS data; ICD-10 codes: C00-D48) **or** hospitalization during follow-up with main diagnosis of radiotherapy (Z51.0) or chemotherapy (Z51.1) + related diagnosis in relation to cancer (SNDS data; ICD-10 codes: C00-D48) |
| Use of antidepressants | ≥ 1 dispensing of antidepressants in the 6 months following inclusion (SNDS data; ATC codes: N06A) |
| Use of anxiolytics | ≥ 1 dispensing of anxiolytics in the 6 months following inclusion (SNDS data; ATC codes: N05B) |
| Use of hypnotics | ≥ 1 dispensing of hypnotics in the 6 months following inclusion (SNDS data; ATC codes: N05C) |
| **Occupational exposures (CONSTANCES questionnaires)** | |
| ***Organizational constraints*** | |
| Late hours (bedtime after midnight) | Do you have (or have you had) work and travel times requiring you to go to bed after midnight at least 50 days per year? Y/N |
| Early hours (up before 5am) | Do you have (or have you had) work and travel times requiring you to get up before 5am at least 50 days per year? Y/N |
| Sleepless nights | Do you have (or have you had) work and travel times requiring you not to sleep at night at least 50 days per year? Y/N |
| Long working hours (>10h) | Do you have (or have you had) a daily work time (excluding travel) of more than 10 hours at least 50 days per year? Y/N |
| Weekly rest <48h consecutive | Do you regularly have (or have you had) less than 48 consecutive hours of rest per week? Y/N |
| Shift work | Do you have (or have you had) an alternating times shift-based job (teams, brigades, rotations, etc.)? Y/N |
| Saturday work (more than one in two) | Do you work (or have you worked) more than one in two Saturdays during the year? Y/N |
| Sunday work (more than one in two) | Do you work (or have you worked) more than one in two Sundays during the year? Y/N |
| ***Biomechanical factors*** | |
| Time-constrained job | Do you have (or have you had) a repetitive and time-constrained job (line production work, moving product or parts, automatic rate machine, rate imposed by strict standards, etc.)? Y/N |
| Repetitive work | During a typical working day: Do you need to repeat the same actions more than 2 to 4 times per minute? Likert 1–4 |
| Physically difficult work | During your professional life, have you been (or are you currently) exposed to physically difficult work? Y/N |
| Physical effort at work | For working participants: In your current job, what level of physical effort is required of you?  Physical effort ≥ 3: 0 (sedentary) – 3 (heavy effort) |
| Carry heavy loads | During your professional life, have you been required (or are you currently required) to carry heavy loads? Y/N |
| Carry heavy loads (>25kg) | How much time do you spend carrying a load weighing more than 25 kg? Likert 1–4 |
| Arms above shoulder | During a typical working day, how much time do you have to adopt the following positions: Work with one or both arms raised (above the shoulders) on a regular or prolonged basis? Likert 1–4 |
| Kneel or squat | On a typical working day: Do you need to kneel or crouch? Likert 1–4 |
| Intense physical effort (Borg) | How would you rate the intensity of physical effort during a typical working day?  Borg Rating of Perceived Exertion Scale ≥ 13: 6 (no effort required) – 20 (exhausting) |
| ***Physical and chemical factors*** | |
| Noise pollution | Do you work (or have you worked) in an environment occasionally requiring you to raise your voice to be heard by a person located less than 2 or 3 meters from you? Y/N |
| Noisy tools | Do you work (or have you worked) with or in the vicinity of noisy tools, machines or vehicles? Y/N |
| Ionizing radiation | Are you, or have you been during your professional life, exposed to ionizing radiation (X-rays, gamma rays, etc.)? Y/N |
| Formaldehyde | During your professional life, have you been (or are you currently) exposed to formaldehyde? Y/N |
| ***Psychosocial factors*** | |
| Effort-reward imbalance | *Calculated* – ERI ≥ 1.5 |

a Y/N: Yes/No; Likert 1–4: ‘Never or nearly never’, ‘rarely’, ‘often’, ‘always or nearly always’. ‘Often’ and ‘always or nearly always’ were considered to indicate exposure.

SNDS: French National Healthcare Database (*Système National des Données de Santé*), CES-D: Center for Epidemiologic Studies Depression Scale, ATC: Anatomical Therapeutic Chemical Classification System; ICD-10: International Classification of Diseases - 10^th^ revision, ERI: effort-reward imbalance

**Supplementary material table 2. Association estimates for all tested associations with occupational exposures at baseline**

| **Exposure** | **Outcome** | **Type** | **Unadjusted** | | |  | **Adjusted ^a^** | | |  | **Adjusted ^b^, stratified by sex** | | | | | | |
| --- | --- | --- | --- | --- | --- | --- | --- | --- | --- | --- | --- | --- | --- | --- | --- | --- | --- |
|  |  |  |  |  |  |  |  |  |  |  | **Women** | | |  | **Men** | | |
|  |  |  | **N** | **Event** | **OR [95%CI]** |  | **N** | **Event** | **aOR [95%CI]** |  | **N** | **Event** | **aOR [95%CI]** |  | **N** | **Event** | **aOR [95%CI]** |
| ***Organizational constraints*** |  |  |  |  |  |  |  |  |  |  |  |  |  |  |  |  |  |
| **Late hours (bedtime after midnight)** | **Sleep disorders** | **Self-reported** | **11965** | **774** | **1.08 [0.84;1.36]** |  | **11965** | **774** | **1.32 [1.03;1.67]** |  | **9611** | **686** | **1.42 [1.09;1.83]** |  | **2354** | **88** | **0.86 [0.41;1.61]** |
|  |  | JEM Soignances | 11066 | 711 | 0.89 [0.75;1.04] |  | 11066 | 711 | 0.99 [0.84;1.17] |  | 8896 | 632 | 1.04 [0.88;1.24] |  | 2170 | 79 | 0.66 [0.38;1.09] |
|  |  | Alternative JEM | 8328 | 543 | 1.03 [0.86;1.23] |  | 8328 | 543 | 1.12 [0.93;1.34] |  | 6805 | 488 | 1.15 [0.95;1.39] |  | 1523 | 55 | 0.88 [0.49;1.53] |
| **Late hours (bedtime after midnight)** | **Depressive symptoms** | **Self-reported** | **11718** | **1615** | **0.99 [0.83;1.18]** |  | **11718** | **1615** | **1.07 [0.89;1.27]** |  | **9404** | **1414** | **1.06 [0.87;1.29]** |  | **2314** | **201** | **1.09 [0.71;1.62]** |
|  |  | JEM Soignances | 10843 | 1469 | 0.80 [0.71;0.90] |  | 10843 | 1469 | 0.81 [0.72;0.92] |  | 8708 | 1281 | 0.81 [0.71;0.92] |  | 2135 | 188 | 0.87 [0.62;1.20] |
|  |  | Alternative JEM | 8157 | 1138 | 0.86 [0.75;0.98] |  | 8157 | 1138 | 0.88 [0.77;1.00] |  | 6659 | 1005 | 0.87 [0.76;1.00] |  | 1498 | 133 | 0.94 [0.64;1.35] |
| **Early hours (up before 5am)** | **Sleep disorders** | **Self-reported** | **11962** | **774** | **1.39 [1.08;1.77]** |  | **11962** | **774** | **1.49 [1.15;1.90]** |  | **9611** | **687** | **1.58 [1.21;2.04]** |  | **2351** | **87** | **0.91 [0.35;1.96]** |
|  |  | JEM Soignances | 11066 | 711 | 1.22 [1.05;1.42] |  | 11066 | 711 | 1.20 [1.03;1.40] |  | 8896 | 632 | 1.19 [1.01;1.40] |  | 2170 | 79 | 1.31 [0.83;2.06] |
|  |  | Alternative JEM | 8328 | 543 | 1.55 [1.28;1.86] |  | 8328 | 543 | 1.56 [1.29;1.88] |  | 6805 | 488 | 1.53 [1.24;1.86] |  | 1523 | 55 | 1.85 [1.04;3.23] |
| **Early hours (up before 5am)** | **Depressive symptoms** | **Self-reported** | **11721** | **1614** | **1.56 [1.30;1.86]** |  | **11721** | **1614** | **1.60 [1.33;1.91]** |  | **9408** | **1413** | **1.62 [1.33;1.96]** |  | **2313** | **201** | **1.48 [0.89;2.34]** |
|  |  | JEM Soignances | 10843 | 1469 | 1.01 [0.91;1.13] |  | 10843 | 1469 | 0.99 [0.89;1.11] |  | 8708 | 1281 | 1.02 [0.90;1.14] |  | 2135 | 188 | 0.86 [0.63;1.17] |
|  |  | Alternative JEM | 8157 | 1138 | 1.25 [1.08;1.44] |  | 8157 | 1138 | 1.25 [1.08;1.44] |  | 6659 | 1005 | 1.29 [1.11;1.50] |  | 1498 | 133 | 1.00 [0.64;1.50] |
| **Sleepless nights** | **Sleep disorders** | **Self-reported** | **11999** | **774** | **1.27 [1.01;1.58]** |  | **11999** | **774** | **1.47 [1.16;1.83]** |  | **9644** | **686** | **1.51 [1.18;1.91]** |  | **2355** | **88** | **1.20 [0.57;2.24]** |
|  |  | JEM Soignances | 11066 | 711 | 1.02 [0.87;1.19] |  | 11066 | 711 | 1.13 [0.96;1.32] |  | 8896 | 632 | 1.14 [0.96;1.35] |  | 2170 | 79 | 1.03 [0.64;1.64] |
|  |  | Alternative JEM | 8328 | 543 | 1.24 [1.01;1.50] |  | 8328 | 543 | 1.24 [1.02;1.51] |  | 6805 | 488 | 1.26 [1.02;1.55] |  | 1523 | 55 | 1.04 [0.45;2.12] |
| **Sleepless nights** | **Depressive symptoms** | **Self-reported** | **11758** | **1616** | **0.85 [0.71;1.02]** |  | **11758** | **1616** | **0.88 [0.73;1.05]** |  | **9443** | **1416** | **0.89 [0.72;1.07]** |  | **2315** | **200** | **0.83 [0.48;1.34]** |
|  |  | JEM Soignances | 10843 | 1469 | 0.89 [0.79;0.99] |  | 10843 | 1469 | 0.90 [0.80;1.01] |  | 8708 | 1281 | 0.90 [0.80;1.02] |  | 2135 | 188 | 0.89 [0.64;1.21] |
|  |  | Alternative JEM | 8157 | 1138 | 0.85 [0.73;0.99] |  | 8157 | 1138 | 0.82 [0.70;0.96] |  | 6659 | 1005 | 0.83 [0.70;0.97] |  | 1498 | 133 | 0.78 [0.44;1.30] |
| **Long working hours (>10h)** | **Sleep disorders** | **Self-reported** | **11981** | **780** | **0.79 [0.66;0.94]** |  | **11981** | **780** | **0.91 [0.76;1.09]** |  | **9620** | **692** | **0.94 [0.77;1.14]** |  | **2361** | **88** | **0.79 [0.49;1.23]** |
|  |  | JEM Soignances | 11066 | 711 | 0.60 [0.50;0.73] |  | 11066 | 711 | 0.67 [0.55;0.81] |  | 8896 | 632 | 0.75 [0.60;0.91] |  | 2170 | 79 | 0.36 [0.20;0.60] |
|  |  | Alternative JEM | 8317 | 542 | 0.78 [0.64;0.95] |  | 8317 | 542 | 0.89 [0.73;1.08] |  | 6796 | 487 | 0.93 [0.75;1.14] |  | 1521 | 55 | 0.66 [0.37;1.14] |
| **Long working hours (>10h)** | **Depressive symptoms** | **Self-reported** | **11740** | **1617** | **0.85 [0.75;0.96]** |  | **11740** | **1617** | **0.92 [0.81;1.04]** |  | **9419** | **1416** | **0.93 [0.81;1.07]** |  | **2321** | **201** | **0.89 [0.65;1.20]** |
|  |  | JEM Soignances | 10843 | 1469 | 0.60 [0.52;0.68] |  | 10843 | 1469 | 0.64 [0.56;0.74] |  | 8708 | 1281 | 0.64 [0.55;0.74] |  | 2135 | 188 | 0.65 [0.47;0.88] |
|  |  | Alternative JEM | 8146 | 1137 | 0.66 [0.57;0.76] |  | 8146 | 1137 | 0.71 [0.62;0.82] |  | 6650 | 1004 | 0.70 [0.59;0.81] |  | 1496 | 133 | 0.80 [0.56;1.15] |
| **Weekly rest < 48h consecutive** | **Sleep disorders** | **Self-reported** | **11921** | **775** | **1.25 [1.03;1.50]** |  | **11921** | **775** | **1.41 [1.16;1.70]** |  | **9569** | **687** | **1.54 [1.26;1.88]** |  | **2352** | **88** | **0.73 [0.37;1.31]** |
|  |  | JEM Soignances | 11056 | 710 | 1.16 [0.99;1.36] |  | 11056 | 710 | 1.21 [1.03;1.43] |  | 8886 | 631 | 1.27 [1.07;1.52] |  | 2170 | 79 | 0.88 [0.56;1.40] |
|  |  | Alternative JEM | 8328 | 543 | 1.12 [0.94;1.33] |  | 8328 | 543 | 1.19 [1.00;1.43] |  | 6805 | 488 | 1.21 [1.00;1.45] |  | 1523 | 55 | 1.10 [0.64;1.90] |
| **Weekly rest < 48h consecutive** | **Depressive symptoms** | **Self-reported** | **11683** | **1612** | **1.30 [1.13;1.49]** |  | **11683** | **1612** | **1.36 [1.19;1.56]** |  | **9371** | **1411** | **1.40 [1.21;1.63]** |  | **2312** | **201** | **1.17 [0.81;1.66]** |
|  |  | JEM Soignances | 10833 | 1467 | 1.26 [1.12;1.42] |  | 10833 | 1467 | 1.27 [1.13;1.43] |  | 8698 | 1279 | 1.32 [1.16;1.50] |  | 2135 | 188 | 1.02 [0.75;1.39] |
|  |  | Alternative JEM | 8157 | 1138 | 1.21 [1.07;1.37] |  | 8157 | 1138 | 1.25 [1.10;1.41] |  | 6659 | 1005 | 1.30 [1.14;1.49] |  | 1498 | 133 | 0.93 [0.65;1.33] |
| **Shift work** | **Sleep disorders** | **Self-reported** | **11910** | **772** | **1.31 [1.11;1.55]** |  | **11910** | **772** | **1.49 [1.25;1.76]** |  | **9565** | **685** | **1.55 [1.29;1.85]** |  | **2345** | **87** | **1.00 [0.52;1.78]** |
|  |  | JEM Soignances | 11066 | 711 | 1.55 [1.33;1.81] |  | 11066 | 711 | 1.56 [1.34;1.83] |  | 8896 | 632 | 1.50 [1.27;1.76] |  | 2170 | 79 | 2.20 [1.39;3.47] |
|  |  | Alternative JEM | 8306 | 539 | 1.52 [1.28;1.82] |  | 8306 | 539 | 1.50 [1.25;1.79] |  | 6792 | 484 | 1.47 [1.21;1.78] |  | 1514 | 55 | 1.76 [1.02;3.04] |
| **Shift work** | **Depressive symptoms** | **Self-reported** | **11670** | **1603** | **1.29 [1.14;1.45]** |  | **11670** | **1603** | **1.30 [1.15;1.47]** |  | **9365** | **1403** | **1.31 [1.15;1.49]** |  | **2305** | **200** | **1.23 [0.83;1.77]** |
|  |  | JEM Soignances | 10843 | 1469 | 1.34 [1.20;1.49] |  | 10843 | 1469 | 1.29 [1.16;1.44] |  | 8708 | 1281 | 1.29 [1.15;1.45] |  | 2135 | 188 | 1.30 [0.95;1.78] |
|  |  | Alternative JEM | 8135 | 1134 | 1.22 [1.08;1.38] |  | 8135 | 1134 | 1.18 [1.04;1.34] |  | 6646 | 1001 | 1.17 [1.02;1.34] |  | 1489 | 133 | 1.27 [0.88;1.81] |
| **Saturday work (more than one in two)** | **Sleep disorders** | **Self-reported** | **12000** | **779** | **1.35 [1.14;1.58]** |  | **12000** | **779** | **1.46 [1.24;1.72]** |  | **9644** | **691** | **1.51 [1.26;1.79]** |  | **2356** | **88** | **1.17 [0.69;1.91]** |
|  |  | JEM Soignances | 11066 | 711 | 1.46 [1.25;1.71] |  | 11066 | 711 | 1.47 [1.25;1.73] |  | 8896 | 632 | 1.49 [1.26;1.77] |  | 2170 | 79 | 1.33 [0.84;2.09] |
|  |  | Alternative JEM | 8317 | 542 | 1.51 [1.27;1.81] |  | 8317 | 542 | 1.51 [1.26;1.80] |  | 6796 | 487 | 1.55 [1.28;1.87] |  | 1521 | 55 | 1.22 [0.69;2.11] |
| **Saturday work (more than one in two)** | **Depressive symptoms** | **Self-reported** | **11760** | **1620** | **1.32 [1.17;1.49]** |  | **11760** | **1620** | **1.35 [1.19;1.52]** |  | **9444** | **1420** | **1.37 [1.20;1.55]** |  | **2316** | **200** | **1.25 [0.89;1.74]** |
|  |  | JEM Soignances | 10843 | 1469 | 1.28 [1.15;1.43] |  | 10843 | 1469 | 1.24 [1.11;1.39] |  | 8708 | 1281 | 1.27 [1.12;1.43] |  | 2135 | 188 | 1.07 [0.79;1.45] |
|  |  | Alternative JEM | 8146 | 1137 | 1.38 [1.22;1.56] |  | 8146 | 1137 | 1.33 [1.18;1.52] |  | 6650 | 1004 | 1.37 [1.19;1.56] |  | 1496 | 133 | 1.14 [0.78;1.64] |
| **Sunday work (more than one in two)** | **Sleep disorders** | **Self-reported** | **11984** | **777** | **1.56 [1.30;1.85]** |  | **11984** | **777** | **1.64 [1.37;1.96]** |  | **9631** | **689** | **1.68 [1.39;2.02]** |  | **2353** | **88** | **1.31 [0.68;2.31]** |
|  |  | JEM Soignances | 11066 | 711 | 1.67 [1.44;1.95] |  | 11066 | 711 | 1.63 [1.40;1.91] |  | 8896 | 632 | 1.61 [1.37;1.90] |  | 2170 | 79 | 1.82 [1.14;2.88] |
|  |  | Alternative JEM | 8328 | 543 | 1.71 [1.44;2.04] |  | 8328 | 543 | 1.69 [1.42;2.02] |  | 6805 | 488 | 1.72 [1.43;2.08] |  | 1523 | 55 | 1.43 [0.81;2.48] |
| **Sunday work (more than one in two)** | **Depressive symptoms** | **Self-reported** | **11747** | **1617** | **1.46 [1.28;1.67]** |  | **11747** | **1617** | **1.45 [1.27;1.66]** |  | **9433** | **1416** | **1.43 [1.24;1.65]** |  | **2314** | **201** | **1.62 [1.09;2.35]** |
|  |  | JEM Soignances | 10843 | 1469 | 1.35 [1.21;1.51] |  | 10843 | 1469 | 1.28 [1.15;1.43] |  | 8708 | 1281 | 1.29 [1.15;1.46] |  | 2135 | 188 | 1.21 [0.87;1.66] |
|  |  | Alternative JEM | 8157 | 1138 | 1.41 [1.24;1.60] |  | 8157 | 1138 | 1.36 [1.20;1.55] |  | 6659 | 1005 | 1.40 [1.22;1.60] |  | 1498 | 133 | 1.12 [0.76;1.63] |
| ***Biomechanical factors*** |  |  |  |  |  |  |  |  |  |  |  |  |  |  |  |  |  |
| **Time-constrained job** | **Pain** | **Self-reported** | **10241** | **2660** | **1.61 [1.24;2.07]** |  | **10121** | **2628** | **1.77 [1.35;2.32]** |  | **8230** | **2295** | **1.81 [1.34;2.42]** |  | **1891** | **333** | **1.65 [0.83;3.08]** |
|  |  | JEM Soignances | 9478 | 2446 | 2.02 [1.81;2.26] |  | 9364 | 2414 | 1.88 [1.68;2.11] |  | 7623 | 2113 | 1.88 [1.65;2.13] |  | 1741 | 301 | 1.94 [1.44;2.60] |
|  |  | Alternative JEM | 7164 | 1888 | 1.59 [1.40;1.80] |  | 7077 | 1862 | 1.50 [1.32;1.71] |  | 5846 | 1641 | 1.46 [1.27;1.69] |  | 1231 | 221 | 1.68 [1.21;2.33] |
| **Repetitive work** | **Hand pain** | **Self-reported** | **10078** | **511** | **2.41 [2.01;2.89]** |  | **9957** | **506** | **2.20 [1.83;2.64]** |  | **8094** | **454** | **2.27 [1.87;2.76]** |  | **1863** | **52** | **1.66 [0.90;2.94]** |
|  |  | JEM Soignances | 9581 | 484 | 2.18 [1.81;2.62] |  | 9466 | 479 | 2.08 [1.72;2.51] |  | 7713 | 436 | 2.24 [1.84;2.73] |  | 1753 | 43 | 1.05 [0.55;1.94] |
|  |  | Alternative JEM | 7223 | 364 | 2.22 [1.79;2.75] |  | 7134 | 361 | 1.97 [1.58;2.45] |  | 5901 | 330 | 2.05 [1.63;2.58] |  | 1233 | 31 | 1.30 [0.61;2.68] |
| **Repetitive work** | **Pain** | **Self-reported** | **9963** | **2563** | **2.17 [1.97;2.39]** |  | **9844** | **2529** | **2.04 [1.85;2.26]** |  | **7994** | **2204** | **2.12 [1.90;2.36]** |  | **1850** | **325** | **1.64 [1.25;2.14]** |
|  |  | JEM Soignances | 9468 | 2443 | 1.92 [1.75;2.11] |  | 9355 | 2411 | 1.88 [1.70;2.07] |  | 7615 | 2111 | 1.96 [1.76;2.18] |  | 1740 | 300 | 1.47 [1.13;1.90] |
|  |  | Alternative JEM | 7144 | 1883 | 1.95 [1.75;2.17] |  | 7057 | 1857 | 1.84 [1.64;2.05] |  | 5830 | 1636 | 1.91 [1.70;2.16] |  | 1227 | 221 | 1.40 [1.03;1.90] |
| **Physically difficult work** | **Pain** | **Self-reported** | **10182** | **2641** | **1.92 [1.73;2.13]** |  | **10060** | **2608** | **1.82 [1.64;2.03]** |  | **8175** | **2278** | **1.85 [1.65;2.08]** |  | **1885** | **330** | **1.64 [1.20;2.21]** |
|  |  | JEM Soignances | 9468 | 2443 | 1.74 [1.59;1.91] |  | 9355 | 2411 | 1.56 [1.41;1.72] |  | 7615 | 2111 | 1.54 [1.39;1.71] |  | 1740 | 300 | 1.71 [1.32;2.23] |
|  |  | Alternative JEM | 7154 | 1886 | 1.64 [1.48;1.82] |  | 7067 | 1860 | 1.49 [1.33;1.66] |  | 5838 | 1639 | 1.47 [1.30;1.65] |  | 1229 | 221 | 1.65 [1.22;2.24] |
| **Physical effort at work** | **Pain** | **Self-reported** | **9947** | **2564** | **1.97 [1.75;2.21]** |  | **9829** | **2533** | **1.96 [1.73;2.21]** |  | **7969** | **2200** | **1.95 [1.71;2.22]** |  | **1860** | **333** | **1.99 [1.45;2.72]** |
|  |  | JEM Soignances | 9459 | 2442 | 1.63 [1.49;1.79] |  | 9346 | 2410 | 1.52 [1.38;1.68] |  | 7609 | 2110 | 1.56 [1.41;1.73] |  | 1737 | 300 | 1.33 [1.03;1.73] |
|  |  | Alternative JEM | 7130 | 1876 | 1.46 [1.31;1.63] |  | 7044 | 1850 | 1.38 [1.23;1.54] |  | 5826 | 1634 | 1.40 [1.24;1.58] |  | 1218 | 216 | 1.27 [0.94;1.73] |
| **Carry heavy loads** | **Low back pain** | **Self-reported** | **10465** | **1387** | **1.89 [1.68;2.13]** |  | **10336** | **1367** | **1.84 [1.63;2.08]** |  | **8382** | **1196** | **1.83 [1.61;2.09]** |  | **1954** | **171** | **1.91 [1.34;2.69]** |
|  |  | JEM Soignances | 9726 | 1280 | 1.69 [1.50;1.90] |  | 9605 | 1260 | 1.53 [1.35;1.73] |  | 7808 | 1109 | 1.55 [1.36;1.77] |  | 1797 | 151 | 1.42 [1.00;2.00] |
|  |  | Alternative JEM | 7336 | 1003 | 1.52 [1.33;1.75] |  | 7242 | 987 | 1.41 [1.22;1.62] |  | 5976 | 877 | 1.43 [1.23;1.67] |  | 1266 | 110 | 1.26 [0.85;1.88] |
| **Carry heavy loads** | **Pain** | **Self-reported** | **10203** | **2649** | **1.69 [1.53;1.86]** |  | **10081** | **2616** | **1.69 [1.52;1.87]** |  | **8187** | **2281** | **1.69 [1.52;1.89]** |  | **1894** | **335** | **1.66 [1.25;2.18]** |
|  |  | JEM Soignances | 9478 | 2446 | 1.58 [1.44;1.73] |  | 9364 | 2414 | 1.45 [1.31;1.60] |  | 7623 | 2113 | 1.44 [1.30;1.60] |  | 1741 | 301 | 1.53 [1.17;1.98] |
|  |  | Alternative JEM | 7154 | 1886 | 1.43 [1.29;1.59] |  | 7067 | 1860 | 1.33 [1.19;1.49] |  | 5838 | 1639 | 1.31 [1.16;1.48] |  | 1229 | 221 | 1.48 [1.09;2.00] |
| **Carry heavy loads (>25kg)** | **Low back pain** | **Self-reported** | **10166** | **1341** | **2.66 [2.32;3.05]** |  | **10043** | **1320** | **2.48 [2.16;2.86]** |  | **8138** | **1154** | **2.42 [2.08;2.81]** |  | **1905** | **166** | **3.01 [2.02;4.41]** |
|  |  | JEM Soignances | 9726 | 1280 | 2.68 [2.34;3.06] |  | 9605 | 1260 | 2.38 [2.07;2.73] |  | 7808 | 1109 | 2.44 [2.10;2.82] |  | 1797 | 151 | 2.00 [1.32;2.98] |
|  |  | Alternative JEM | 7299 | 999 | 2.30 [1.98;2.67] |  | 7206 | 983 | 2.07 [1.77;2.42] |  | 5947 | 874 | 2.17 [1.84;2.56] |  | 1259 | 109 | 1.42 [0.86;2.26] |
| **Carry heavy loads (>25kg)** | **Pain** | **Self-reported** | **9923** | **2567** | **2.36 [2.10;2.65]** |  | **9807** | **2534** | **2.24 [1.98;2.53]** |  | **7958** | **2212** | **2.20 [1.93;2.51]** |  | **1849** | **322** | **2.49 [1.79;3.45]** |
|  |  | JEM Soignances | 9478 | 2446 | 2.46 [2.20;2.76] |  | 9364 | 2414 | 2.24 [1.99;2.52] |  | 7623 | 2113 | 2.28 [2.01;2.59] |  | 1741 | 301 | 2.01 [1.45;2.77] |
|  |  | Alternative JEM | 7118 | 1873 | 2.22 [1.95;2.52] |  | 7032 | 1847 | 2.02 [1.77;2.31] |  | 5809 | 1628 | 2.09 [1.81;2.41] |  | 1223 | 219 | 1.66 [1.14;2.38] |
| **Arms above shoulder** | **Shoulder pain** | **Self-reported** | **10274** | **784** | **2.62 [2.16;3.15]** |  | **10151** | **778** | **2.37 [1.94;2.87]** |  | **8256** | **691** | **2.17 [1.76;2.65]** |  | **1895** | **87** | **6.00 [3.21;10.8]** |
|  |  | JEM Soignances | 9651 | 745 | 1.98 [1.68;2.32] |  | 9531 | 738 | 1.80 [1.52;2.12] |  | 7764 | 659 | 1.73 [1.45;2.05] |  | 1767 | 79 | 2.56 [1.54;4.17] |
|  |  | Alternative JEM | 7262 | 570 | 1.63 [1.35;1.96] |  | 7170 | 565 | 1.53 [1.26;1.84] |  | 5928 | 516 | 1.48 [1.21;1.80] |  | 1242 | 49 | 2.13 [1.13;3.90] |
| **Arms above shoulder** | **Pain** | **Self-reported** | **10098** | **2622** | **2.03 [1.77;2.33]** |  | **9981** | **2589** | **1.84 [1.60;2.12]** |  | **8111** | **2260** | **1.78 [1.53;2.06]** |  | **1870** | **329** | **2.66 [1.65;4.21]** |
|  |  | JEM Soignances | 9478 | 2446 | 2.03 [1.82;2.25] |  | 9364 | 2414 | 1.91 [1.72;2.13] |  | 7623 | 2113 | 1.94 [1.73;2.18] |  | 1741 | 301 | 1.74 [1.27;2.37] |
|  |  | Alternative JEM | 7135 | 1880 | 1.54 [1.36;1.73] |  | 7049 | 1854 | 1.47 [1.30;1.66] |  | 5824 | 1635 | 1.46 [1.28;1.66] |  | 1225 | 219 | 1.56 [1.09;2.21] |
| **Kneel or squat** | **Knee pain** | **Self-reported** | **10308** | **743** | **2.16 [1.86;2.52]** |  | **10186** | **732** | **2.28 [1.95;2.67]** |  | **8272** | **620** | **2.27 [1.92;2.69]** |  | **1914** | **112** | **2.38 [1.57;3.59]** |
|  |  | JEM Soignances | 9620 | 678 | 1.89 [1.58;2.28] |  | 9505 | 668 | 1.85 [1.53;2.24] |  | 7722 | 562 | 1.81 [1.47;2.25] |  | 1783 | 106 | 2.00 [1.32;3.10] |
|  |  | Alternative JEM | 7250 | 527 | 2.44 [2.03;2.94] |  | 7162 | 520 | 2.25 [1.86;2.72] |  | 5908 | 440 | 2.29 [1.86;2.83] |  | 1254 | 80 | 2.06 [1.29;3.28] |
| **Kneel or squat** | **Pain** | **Self-reported** | **10162** | **2640** | **1.94 [1.77;2.12]** |  | **10041** | **2606** | **1.95 [1.78;2.15]** |  | **8171** | **2276** | **1.96 [1.77;2.17]** |  | **1870** | **330** | **1.88 [1.43;2.47]** |
|  |  | JEM Soignances | 9478 | 2446 | 1.66 [1.50;1.84] |  | 9364 | 2414 | 1.59 [1.43;1.76] |  | 7623 | 2113 | 1.58 [1.40;1.77] |  | 1741 | 301 | 1.67 [1.28;2.18] |
|  |  | Alternative JEM | 7143 | 1884 | 2.03 [1.83;2.26] |  | 7056 | 1858 | 1.87 [1.67;2.08] |  | 5828 | 1637 | 1.90 [1.68;2.14] |  | 1228 | 221 | 1.68 [1.23;2.29] |
| **Intense physical effort (Borg)** | **Low back pain** | **Self-reported** | **10336** | **1367** | **2.35 [2.08;2.66]** |  | **10211** | **1348** | **2.20 [1.94;2.49]** |  | **8293** | **1179** | **2.25 [1.97;2.58]** |  | **1918** | **169** | **1.91 [1.38;2.66]** |
|  |  | JEM Soignances | 9716 | 1277 | 1.65 [1.45;1.89] |  | 9596 | 1257 | 1.55 [1.35;1.77] |  | 7800 | 1107 | 1.56 [1.35;1.80] |  | 1796 | 150 | 1.48 [1.04;2.14] |
|  |  | Alternative JEM | 7328 | 1003 | 1.54 [1.33;1.79] |  | 7235 | 987 | 1.41 [1.21;1.65] |  | 5970 | 878 | 1.44 [1.22;1.69] |  | 1265 | 109 | 1.30 [0.86;1.99] |
| **Intense physical effort (Borg)** | **Pain** | **Self-reported** | **10080** | **2615** | **2.09 [1.91;2.29]** |  | **9961** | **2583** | **1.99 [1.81;2.19]** |  | **8103** | **2255** | **2.02 [1.82;2.24]** |  | **1858** | **328** | **1.84 [1.44;2.36]** |
|  |  | JEM Soignances | 9468 | 2443 | 1.54 [1.39;1.70] |  | 9355 | 2411 | 1.46 [1.32;1.62] |  | 7615 | 2111 | 1.47 [1.32;1.65] |  | 1740 | 300 | 1.43 [1.10;1.88] |
|  |  | Alternative JEM | 7146 | 1882 | 1.46 [1.31;1.64] |  | 7060 | 1856 | 1.36 [1.21;1.54] |  | 5832 | 1637 | 1.37 [1.20;1.56] |  | 1228 | 219 | 1.34 [0.98;1.85] |
| ***Physical and chemical factors*** |  |  |  |  |  |  |  |  |  |  |  |  |  |  |  |  |  |
| **Noise pollution** | **Hypertension** | **Self-reported** | **12140** | **2087** | **0.99 [0.84;1.16]** |  | **12140** | **2087** | **1.20 [1.01;1.42]** |  | **9758** | **1452** | **1.31 [1.08;1.58]** |  | **2382** | **635** | **0.83 [0.55;1.22]** |
|  |  | JEM Soignances | 11192 | 1894 | 1.22 [1.07;1.39] |  | 11192 | 1894 | 1.12 [0.97;1.29] |  | 8999 | 1310 | 1.14 [0.97;1.35] |  | 2193 | 584 | 1.07 [0.82;1.39] |
|  |  | Alternative JEM | 8412 | 1420 | 1.18 [1.02;1.37] |  | 8412 | 1420 | 1.08 [0.92;1.26] |  | 6879 | 1011 | 1.08 [0.90;1.29] |  | 1533 | 409 | 1.08 [0.79;1.45] |
| **Noise pollution** | **Depressive symptoms** | **Self-reported** | 11763 | 1619 | **1.75 [1.49;2.05]** |  | 11763 | 1619 | **1.74 [1.48;2.04]** |  | 9445 | 1418 | **1.72 [1.45;2.03]** |  | 2318 | 201 | **1.94 [1.20;3.03]** |
|  |  | JEM Soignances | 10843 | 1469 | 0.98 [0.84;1.15] |  | 10843 | 1469 | 0.99 [0.84;1.15] |  | 8708 | 1281 | 1.00 [0.84;1.18] |  | 2135 | 188 | 0.93 [0.61;1.39] |
|  |  | Alternative JEM | 8157 | 1138 | 1.17 [0.99;1.37] |  | 8157 | 1138 | 1.16 [0.98;1.36] |  | 6659 | 1005 | 1.20 [1.01;1.43] |  | 1498 | 133 | 0.89 [0.54;1.42] |
| **Noisy tools** | **Hypertension** | **Self-reported** | **12116** | **2080** | **1.02 [0.84;1.23]** |  | **12116** | **2080** | **1.11 [0.90;1.35]** |  | **9736** | **1447** | **1.13 [0.88;1.44]** |  | **2380** | **633** | **1.04 [0.72;1.48]** |
|  |  | JEM Soignances | 11192 | 1894 | 1.18 [1.00;1.38] |  | 11192 | 1894 | 1.03 [0.87;1.22] |  | 8999 | 1310 | 0.97 [0.78;1.20] |  | 2193 | 584 | 1.15 [0.87;1.52] |
|  |  | Alternative JEM | 8412 | 1420 | 1.14 [0.94;1.37] |  | 8412 | 1420 | 1.06 [0.87;1.28] |  | 6879 | 1011 | 0.96 [0.75;1.21] |  | 1533 | 409 | 1.28 [0.91;1.79] |
| **Noisy tools** | **Depressive symptoms** | **Self-reported** | 11739 | 1615 | **1.13 [0.91;1.38]** |  | 11739 | 1615 | **1.18 [0.95;1.45]** |  | 9423 | 1412 | **1.18 [0.93;1.48]** |  | 2316 | 203 | **1.16 [0.68;1.89]** |
|  |  | JEM Soignances | 10843 | 1469 | 0.85 [0.69;1.02] |  | 10843 | 1469 | 0.88 [0.72;1.07] |  | 8708 | 1281 | 0.87 [0.69;1.08] |  | 2135 | 188 | 0.96 [0.61;1.46] |
|  |  | Alternative JEM | 8157 | 1138 | 0.79 [0.63;0.99] |  | 8157 | 1138 | 0.81 [0.64;1.02] |  | 6659 | 1005 | 0.89 [0.69;1.12] |  | 1498 | 133 | 0.46 [0.21;0.87] |
| **Ionizing radiation** | **Cancer** | **Self-reported** | **11035** | **921** | **1.05 [0.86;1.27]** |  | **11035** | **921** | **1.28 [1.04;1.56]** |  | **8824** | **775** | **1.22 [0.96;1.53]** |  | **2211** | **146** | **1.39 [0.91;2.09]** |
|  |  | JEM Soignances | 10521 | 883 | 0.92 [0.73;1.15] |  | 10521 | 883 | 1.08 [0.85;1.35] |  | 8418 | 747 | 1.04 [0.79;1.34] |  | 2103 | 136 | 1.21 [0.74;1.93] |
|  |  | Alternative JEM | 7918 | 640 | 0.78 [0.60;1.01] |  | 7918 | 640 | 0.90 [0.68;1.17] |  | 6448 | 553 | 0.93 [0.69;1.24] |  | 1470 | 87 | 0.80 [0.39;1.49] |
| **Formaldehyde** | **Cancer** | **Self-reported** | **11124** | **941** | **1.04 [0.68;1.52]** |  | **11124** | **941** | **1.18 [0.77;1.74]** |  | **8886** | **796** | **1.14 [0.72;1.74]** |  | **2238** | **145** | **1.56 [0.45;4.08]** |
|  |  | JEM Soignances | 10578 | 891 | 0.92 [0.58;1.39] |  | 10578 | 891 | 0.94 [0.59;1.42] |  | 8458 | 755 | 0.94 [0.56;1.48] |  | 2120 | 136 | 0.95 [0.28;2.41] |
|  |  | Alternative JEM | 7916 | 635 | 0.85 [0.50;1.37] |  | 7916 | 635 | 0.84 [0.49;1.36] |  | 6440 | 548 | 0.82 [0.45;1.38] |  | 1476 | 87 | 0.97 [0.23;2.81] |
| ***Psychosocial factor*** |  |  |  |  |  |  |  |  |  |  |  |  |  |  |  |  |  |
| **Effort-reward imbalance** | **Depressive symptoms** | **Self-reported** | **11481** | **1567** | **3.27 [2.89;3.69]** |  | **11481** | **1567** | **3.18 [2.81;3.59]** |  | **9212** | **1371** | **3.15 [2.77;3.59]** |  | **2269** | **196** | **3.37 [2.36;4.77]** |
|  |  | JEM Soignances | 10821 | 1467 | 1.43 [1.28;1.60] |  | 10821 | 1467 | 1.37 [1.23;1.54] |  | 8690 | 1279 | 1.43 [1.27;1.62] |  | 2131 | 188 | 1.06 [0.78;1.44] |
|  |  | Alternative JEM | 8102 | 1129 | 1.04 [0.91;1.18] |  | 8102 | 1129 | 0.99 [0.87;1.13] |  | 6627 | 997 | 0.98 [0.86;1.13] |  | 1475 | 132 | 1.04 [0.69;1.53] |
| **Effort-reward imbalance** | **Antidepressants** | **Self-reported** | **11781** | **769** | **2.11 [1.78;2.50]** |  | **11781** | **769** | **2.07 [1.74;2.45]** |  | **9459** | **666** | **2.05 [1.71;2.46]** |  | **2322** | **103** | **2.17 [1.29;3.51]** |
|  |  | JEM Soignances | 11177 | 741 | 1.29 [1.11;1.50] |  | 11177 | 741 | 1.26 [1.08;1.47] |  | 8986 | 642 | 1.31 [1.11;1.55] |  | 2191 | 99 | 0.96 [0.63;1.44] |
|  |  | Alternative JEM | 8360 | 578 | 0.99 [0.83;1.17] |  | 8360 | 578 | 0.94 [0.79;1.12] |  | 6849 | 512 | 0.97 [0.80;1.16] |  | 1511 | 66 | 0.70 [0.37;1.24] |
| **Effort-reward imbalance** | **Anxiolytics** | **Self-reported** | **11781** | **839** | **1.79 [1.51;2.11]** |  | **11781** | **839** | **1.73 [1.46;2.04]** |  | **9459** | **741** | **1.70 [1.42;2.02]** |  | **2322** | **98** | **2.05 [1.19;3.39]** |
|  |  | JEM Soignances | 11177 | 814 | 1.14 [0.99;1.32] |  | 11177 | 814 | 1.09 [0.94;1.26] |  | 8986 | 723 | 1.09 [0.93;1.27] |  | 2191 | 91 | 1.07 [0.70;1.64] |
|  |  | Alternative JEM | 8360 | 602 | 1.02 [0.86;1.21] |  | 8360 | 602 | 0.97 [0.81;1.14] |  | 6849 | 542 | 0.97 [0.81;1.16] |  | 1511 | 60 | 0.87 [0.46;1.54] |
| **Effort-reward imbalance** | **Hypnotics** | **Self-reported** | **11781** | **342** | **1.27 [0.95;1.66]** |  | **11781** | **342** | **1.28 [0.96;1.68]** |  | **9459** | **273** | **1.34 [0.99;1.80]** |  | **2322** | **69** | **0.94 [0.39;1.95]** |
|  |  | JEM Soignances | 11177 | 326 | 1.03 [0.82;1.28] |  | 11177 | 326 | 1.07 [0.85;1.34] |  | 8986 | 259 | 1.13 [0.88;1.47] |  | 2191 | 67 | 0.82 [0.49;1.36] |
|  |  | Alternative JEM | 8360 | 236 | 1.09 [0.83;1.41] |  | 8360 | 236 | 1.07 [0.82;1.39] |  | 6849 | 196 | 1.08 [0.81;1.43] |  | 1511 | 40 | 1.02 [0.48;2.01] |

^a^ Adjusted for age and sex (+BMI for biomechanical factors)

^b^ Adjusted for age (+BMI for biomechanical factors)

aOR: adjusted odds ratio, OR: odds ratio, 95%CI: 95% confidence interval

**Supplementary material table 3. Association estimates for all tested associations with cumulative time of occupational exposures (≥10y) at baseline**

| **Exposure** | **Outcome** | **Type** | **Unadjusted** | | |  | **Adjusted ^a^** | | |  | **Adjusted ^b^, stratified by sex** | | | | | | |
| --- | --- | --- | --- | --- | --- | --- | --- | --- | --- | --- | --- | --- | --- | --- | --- | --- | --- |
|  |  |  |  |  |  |  |  |  |  |  | **Women** | | |  | **Men** | | |
|  |  |  | **N** | **Event** | **OR [95%CI]** |  | **N** | **Event** | **aOR [95%CI]** |  | **N** | **Event** | **aOR [95%CI]** |  | **N** | **Event** | **aOR [95%CI]** |
| ***Organizational constraints*** |  |  |  |  |  |  |  |  |  |  |  |  |  |  |  |  |  |
| **Late hours (bedtime after midnight)** | **Sleep disorders** | **Self-reported** | **11965** | **774** | **1.38 [1.08;1.73]** |  | **11965** | **774** | **1.21 [0.95;1.52]** |  | **9611** | **686** | **1.30 [1.01;1.67]** |  | **2354** | **88** | **0.77 [0.37;1.46]** |
|  |  | JEM Soignances | 12039 | 787 | 1.25 [1.07;1.47] |  | 12039 | 787 | 1.00 [0.85;1.18] |  | 9678 | 699 | 1.03 [0.87;1.22] |  | 2361 | 88 | 0.75 [0.42;1.26] |
| **Late hours (bedtime after midnight)** | **Depressive symptoms** | **Self-reported** | **11718** | **1615** | **1.13 [0.94;1.35]** |  | **11718** | **1615** | **1.13 [0.94;1.36]** |  | **9404** | **1414** | **1.14 [0.92;1.39]** |  | **2314** | **201** | **1.15 [0.73;1.75]** |
|  |  | JEM Soignances | 11798 | 1630 | 0.88 [0.78;1.00] |  | 11798 | 1630 | 0.81 [0.71;0.92] |  | 9477 | 1427 | 0.78 [0.68;0.90] |  | 2321 | 203 | 1.00 [0.69;1.42] |
| **Early hours (up before 5am)** | **Sleep disorders** | **Self-reported** | **11962** | **774** | **1.65 [1.29;2.09]** |  | **11962** | **774** | **1.37 [1.07;1.75]** |  | **9611** | **687** | **1.41 [1.08;1.82]** |  | **2351** | **87** | **1.14 [0.50;2.27]** |
|  |  | JEM Soignances | 12039 | 787 | 1.44 [1.24;1.67] |  | 12039 | 787 | 1.11 [0.95;1.30] |  | 9678 | 699 | 1.08 [0.92;1.27] |  | 2361 | 88 | 1.36 [0.85;2.14] |
| **Early hours (up before 5am)** | **Depressive symptoms** | **Self-reported** | **11721** | **1614** | **1.53 [1.27;1.84]** |  | **11721** | **1614** | **1.49 [1.23;1.79]** |  | **9408** | **1413** | **1.47 [1.19;1.79]** |  | **2313** | **201** | **1.61 [0.96;2.58]** |
|  |  | JEM Soignances | 11798 | 1630 | 1.05 [0.93;1.17] |  | 11798 | 1630 | 0.96 [0.85;1.08] |  | 9477 | 1427 | 0.94 [0.83;1.07] |  | 2321 | 203 | 1.05 [0.75;1.46] |
| **Sleepless nights** | **Sleep disorders** | **Self-reported** | **11999** | **774** | **1.62 [1.30;2.00]** |  | **11999** | **774** | **1.33 [1.06;1.65]** |  | **9644** | **686** | **1.37 [1.08;1.72]** |  | **2355** | **88** | **1.09 [0.52;2.06]** |
|  |  | JEM Soignances | 12039 | 787 | 1.35 [1.16;1.58] |  | 12039 | 787 | 1.07 [0.91;1.25] |  | 9678 | 699 | 1.06 [0.90;1.26] |  | 2361 | 88 | 1.09 [0.66;1.76] |
| **Sleepless nights** | **Depressive symptoms** | **Self-reported** | **11758** | **1616** | **1.15 [0.96;1.36]** |  | **11758** | **1616** | **1.09 [0.91;1.30]** |  | **9443** | **1416** | **1.10 [0.91;1.33]** |  | **2315** | **200** | **1.04 [0.61;1.68]** |
|  |  | JEM Soignances | 11798 | 1630 | 0.91 [0.81;1.03] |  | 11798 | 1630 | 0.83 [0.74;0.94] |  | 9477 | 1427 | 0.83 [0.72;0.94] |  | 2321 | 203 | 0.87 [0.60;1.25] |
| **Long working hours (>10h)** | **Sleep disorders** | **Self-reported** | **11981** | **780** | **1.10 [0.91;1.33]** |  | **11981** | **780** | **0.96 [0.78;1.16]** |  | **9620** | **692** | **1.03 [0.83;1.26]** |  | **2361** | **88** | **0.64 [0.37;1.08]** |
|  |  | JEM Soignances | 12039 | 787 | 0.74 [0.60;0.91] |  | 12039 | 787 | 0.61 [0.49;0.76] |  | 9678 | 699 | 0.65 [0.52;0.82] |  | 2361 | 88 | 0.44 [0.24;0.76] |
| **Long working hours (>10h)** | **Depressive symptoms** | **Self-reported** | **11740** | **1617** | **0.97 [0.84;1.12]** |  | **11740** | **1617** | **0.98 [0.85;1.14]** |  | **9419** | **1416** | **1.03 [0.87;1.21]** |  | **2321** | **201** | **0.80 [0.55;1.14]** |
|  |  | JEM Soignances | 11798 | 1630 | 0.70 [0.60;0.81] |  | 11798 | 1630 | 0.69 [0.59;0.81] |  | 9477 | 1427 | 0.70 [0.58;0.83] |  | 2321 | 203 | 0.68 [0.46;0.97] |
| **Weekly rest < 48h consecutive** | **Sleep disorders** | **Self-reported** | **11921** | **775** | **1.42 [1.17;1.73]** |  | **11921** | **775** | **1.19 [0.97;1.45]** |  | **9569** | **687** | **1.30 [1.05;1.60]** |  | **2352** | **88** | **0.61 [0.30;1.13]** |
|  |  | JEM Soignances | 12039 | 787 | 1.38 [1.20;1.60] |  | 12039 | 787 | 1.03 [0.89;1.20] |  | 9678 | 699 | 1.03 [0.88;1.21] |  | 2361 | 88 | 1.02 [0.65;1.61] |
| **Weekly rest < 48h consecutive** | **Depressive symptoms** | **Self-reported** | **11683** | **1612** | **1.28 [1.10;1.48]** |  | **11683** | **1612** | **1.25 [1.07;1.45]** |  | **9371** | **1411** | **1.26 [1.07;1.49]** |  | **2312** | **201** | **1.21 [0.80;1.79]** |
|  |  | JEM Soignances | 11798 | 1630 | 1.14 [1.02;1.26] |  | 11798 | 1630 | 1.05 [0.94;1.17] |  | 9477 | 1427 | 1.03 [0.92;1.17] |  | 2321 | 203 | 1.15 [0.84;1.58] |
| **Shift work** | **Sleep disorders** | **Self-reported** | **11910** | **772** | **1.60 [1.35;1.89]** |  | **11910** | **772** | **1.33 [1.12;1.58]** |  | **9565** | **685** | **1.30 [1.08;1.56]** |  | **2345** | **87** | **1.62 [0.91;2.73]** |
|  |  | JEM Soignances | 12039 | 787 | 1.73 [1.49;2.00] |  | 12039 | 787 | 1.35 [1.16;1.57] |  | 9678 | 699 | 1.30 [1.10;1.52] |  | 2361 | 88 | 1.80 [1.12;2.82] |
| **Shift work** | **Depressive symptoms** | **Self-reported** | **11670** | **1603** | **1.34 [1.18;1.53]** |  | **11670** | **1603** | **1.25 [1.10;1.43]** |  | **9365** | **1403** | **1.24 [1.08;1.43]** |  | **2305** | **200** | **1.33 [0.87;1.98]** |
|  |  | JEM Soignances | 11798 | 1630 | 1.25 [1.12;1.39] |  | 11798 | 1630 | 1.14 [1.01;1.28] |  | 9477 | 1427 | 1.11 [0.98;1.25] |  | 2321 | 203 | 1.36 [0.96;1.90] |
| **Saturday work (more than one in two)** | **Sleep disorders** | **Self-reported** | **12000** | **779** | **1.58 [1.34;1.85]** |  | **12000** | **779** | **1.29 [1.09;1.52]** |  | **9644** | **691** | **1.35 [1.13;1.61]** |  | **2356** | **88** | **0.89 [0.51;1.49]** |
|  |  | JEM Soignances | 12039 | 787 | 1.56 [1.35;1.81] |  | 12039 | 787 | 1.19 [1.02;1.39] |  | 9678 | 699 | 1.17 [1.00;1.37] |  | 2361 | 88 | 1.39 [0.87;2.19] |
| **Saturday work (more than one in two)** | **Depressive symptoms** | **Self-reported** | **11760** | **1620** | **1.36 [1.20;1.54]** |  | **11760** | **1620** | **1.32 [1.16;1.50]** |  | **9444** | **1420** | **1.34 [1.17;1.53]** |  | **2316** | **200** | **1.20 [0.82;1.72]** |
|  |  | JEM Soignances | 11798 | 1630 | 1.19 [1.07;1.33] |  | 11798 | 1630 | 1.08 [0.97;1.21] |  | 9477 | 1427 | 1.07 [0.94;1.20] |  | 2321 | 203 | 1.19 [0.85;1.66] |
| **Sunday work (more than one in two)** | **Sleep disorders** | **Self-reported** | **11984** | **777** | **1.80 [1.50;2.14]** |  | **11984** | **777** | **1.47 [1.23;1.76]** |  | **9631** | **689** | **1.49 [1.23;1.80]** |  | **2353** | **88** | **1.32 [0.70;2.32]** |
|  |  | JEM Soignances | 12039 | 787 | 1.71 [1.47;1.98] |  | 12039 | 787 | 1.32 [1.13;1.54] |  | 9678 | 699 | 1.30 [1.10;1.52] |  | 2361 | 88 | 1.54 [0.93;2.47] |
| **Sunday work (more than one in two)** | **Depressive symptoms** | **Self-reported** | **11747** | **1617** | **1.51 [1.32;1.73]** |  | **11747** | **1617** | **1.43 [1.24;1.65]** |  | **9433** | **1416** | **1.45 [1.25;1.68]** |  | **2314** | **201** | **1.28 [0.81;1.95]** |
|  |  | JEM Soignances | 11798 | 1630 | 1.26 [1.12;1.40] |  | 11798 | 1630 | 1.13 [1.01;1.27] |  | 9477 | 1427 | 1.11 [0.98;1.26] |  | 2321 | 203 | 1.28 [0.88;1.82] |
| ***Biomechanical factors*** |  |  |  |  |  |  |  |  |  |  |  |  |  |  |  |  |  |
| **Time-constrained job** | **Pain** | **Self-reported** | **10241** | **2660** | **2.20 [1.64;2.94]** |  | **10121** | **2628** | **2.02 [1.49;2.72]** |  | **8230** | **2295** | **1.88 [1.34;2.63]** |  | **1891** | **333** | **2.66 [1.36;5.05]** |
|  |  | JEM Soignances | 10305 | 2688 | 2.12 [1.86;2.41] |  | 10182 | 2654 | 1.68 [1.47;1.92] |  | 8284 | 2317 | 1.69 [1.46;1.96] |  | 1898 | 337 | 1.63 [1.16;2.28] |
| **Physically difficult work** | **Pain** | **Self-reported** | **10182** | **2641** | **2.26 [2.03;2.52]** |  | **10060** | **2608** | **1.76 [1.58;1.98]** |  | **8175** | **2278** | **1.80 [1.59;2.03]** |  | **1885** | **330** | **1.58 [1.15;2.15]** |
|  |  | JEM Soignances | 10305 | 2688 | 1.73 [1.58;1.90] |  | 10182 | 2654 | 1.30 [1.17;1.43] |  | 8284 | 2317 | 1.29 [1.16;1.43] |  | 1898 | 337 | 1.38 [1.04;1.83] |
| **Carry heavy loads** | **Low back pain** | **Self-reported** | **10465** | **1387** | **2.07 [1.83;2.34]** |  | **10336** | **1367** | **1.74 [1.53;1.98]** |  | **8382** | **1196** | **1.74 [1.52;1.99]** |  | **1954** | **171** | **1.81 [1.26;2.55]** |
|  |  | JEM Soignances | 10574 | 1406 | 1.49 [1.33;1.67] |  | 10444 | 1385 | 1.20 [1.06;1.35] |  | 8484 | 1213 | 1.20 [1.06;1.37] |  | 1960 | 172 | 1.21 [0.84;1.71] |
| **Carry heavy loads** | **Pain** | **Self-reported** | **10203** | **2649** | **2.19 [1.98;2.41]** |  | **10081** | **2616** | **1.75 [1.58;1.94]** |  | **8187** | **2281** | **1.77 [1.58;1.99]** |  | **1894** | **335** | **1.64 [1.24;2.17]** |
|  |  | JEM Soignances | 10305 | 2688 | 1.59 [1.45;1.74] |  | 10182 | 2654 | 1.20 [1.09;1.33] |  | 8284 | 2317 | 1.21 [1.09;1.35] |  | 1898 | 337 | 1.18 [0.90;1.55] |
| ***Physical and chemical factors*** |  |  |  |  |  |  |  |  |  |  |  |  |  |  |  |  |  |
| **Noise pollution** | **Hypertension** | **Self-reported** | **12140** | **2087** | **1.27 [1.05;1.52]** |  | **12140** | **2087** | **1.02 [0.83;1.23]** |  | **9758** | **1452** | **1.10 [0.88;1.35]** |  | **2382** | **635** | **0.74 [0.46;1.15]** |
|  |  | JEM Soignances | 12175 | 2086 | 1.54 [1.33;1.78] |  | 12175 | 2086 | 1.05 [0.90;1.22] |  | 9790 | 1451 | 1.10 [0.92;1.31] |  | 2385 | 635 | 0.94 [0.69;1.25] |
| **Noise pollution** | **Depressive symptoms** | **Self-reported** | **11763** | **1619** | **1.83 [1.51;2.21]** |  | **11763** | **1619** | **1.74 [1.43;2.10]** |  | **9445** | **1418** | **1.71 [1.39;2.09]** |  | **2318** | **201** | **1.94 [1.08;3.30]** |
|  |  | JEM Soignances | 11798 | 1630 | 1.02 [0.85;1.21] |  | 11798 | 1630 | 0.98 [0.82;1.17] |  | 9477 | 1427 | 0.97 [0.79;1.17] |  | 2321 | 203 | 1.06 [0.65;1.65] |
| **Noisy tools** | **Hypertension** | **Self-reported** | **12116** | **2080** | **1.40 [1.14;1.71]** |  | **12116** | **2080** | **0.99 [0.80;1.22]** |  | **9736** | **1447** | **1.09 [0.84;1.40]** |  | **2380** | **633** | **0.83 [0.56;1.21]** |
|  |  | JEM Soignances | 12175 | 2086 | 1.43 [1.20;1.69] |  | 12175 | 2086 | 0.96 [0.80;1.15] |  | 9790 | 1451 | 0.95 [0.75;1.19] |  | 2385 | 635 | 1.01 [0.74;1.37] |
| **Noisy tools** | **Depressive symptoms** | **Self-reported** | **11739** | **1615** | **1.22 [0.96;1.54]** |  | **11739** | **1615** | **1.22 [0.96;1.54]** |  | **9423** | **1412** | **1.24 [0.95;1.60]** |  | **2316** | **203** | **1.14 [0.61;1.96]** |
|  |  | JEM Soignances | 11798 | 1630 | 0.93 [0.74;1.15] |  | 11798 | 1630 | 0.94 [0.75;1.16] |  | 9477 | 1427 | 0.89 [0.69;1.13] |  | 2321 | 203 | 1.15 [0.70;1.81] |
| **Ionizing radiation** | **Cancer** | **Self-reported** | **11035** | **921** | **1.51 [1.24;1.84]** |  | **11035** | **921** | **1.24 [1.00;1.51]** |  | **8824** | **775** | **1.19 [0.93;1.50]** |  | **2211** | **146** | **1.30 [0.85;1.95]** |
|  |  | JEM Soignances | 11534 | 964 | 1.36 [1.06;1.73] |  | 11534 | 964 | 0.97 [0.75;1.24] |  | 9224 | 815 | 0.93 [0.69;1.23] |  | 2310 | 149 | 1.02 [0.60;1.66] |
| **Formaldehyde** | **Cancer** | **Self-reported** | **11124** | **941** | **1.33 [0.91;1.90]** |  | **11124** | **941** | **0.92 [0.62;1.32]** |  | **8886** | **796** | **0.93 [0.61;1.37]** |  | **2238** | **145** | **0.94 [0.32;2.23]** |
|  |  | JEM Soignances | 11534 | 964 | 0.99 [0.59;1.56] |  | 11534 | 964 | 0.73 [0.43;1.17] |  | 9224 | 815 | 0.79 [0.45;1.30] |  | 2310 | 149 | 0.51 [0.08;1.71] |

^a^ Adjusted for age and sex (+BMI for biomechanical factors)

^b^ Adjusted for age (+BMI for biomechanical factors)

aOR: adjusted odds ratio, OR: odds ratio, 95%CI: 95% confidence interval
